# A social return on investment analysis of patient-reported outcome measures in value-based healthcare

**DOI:** 10.1186/s41687-025-00853-w

**Published:** 2025-02-20

**Authors:** Ellie Crane, Jane Noyes, Mayara S. Bianchim, Leah Mclaughlin, Adele Cahill, Gareth Roberts, Carys Stringer

**Affiliations:** 1 School of Health Sciences, Bangor University, Bangor, UK; 2 Aneurin Bevan University Health Board, Caerleon, Newport, UK

## Abstract

**Objectives:**

There is growing interest in the use of Patient Reported Outcome Measures (PROMs) to improve patient and healthcare service outcomes. This study aimed to measure the social and economic value of PROMs implemented within a VBHC framework.

**Methods:**

We conducted a Social-Return on Investment (SROI) analysis in Epilepsy, Heart Failure, and Parkinson’s Disease services, to measure the value generated by PROMs for patients and the healthcare provider.

**Results:**

The SROI analysis revealed substantial variation in the value derived from the PROMs intervention across different services. The highest value was observed in Heart Failure with an SROI ratio of 5.55:1, which represents a substantial return on investment for patients and services. In contrast, the Parkinson’s Disease service had small return on investment from PROMs with an SROI ratio of 1.29:1. In Epilepsy, the social value derived from PROMs was proportionally less than the investment made, with an SROI ratio of 0.85:1.

**Conclusion:**

These findings demonstrate the complexities of implementing PROMs within a clinical context, and careful consideration is likely needed in selecting suitable services and tailoring the implementation of PROMs to effectively meet specific service and patient requirements. Where PROMs yielded low or no value, the lack of return-on-investment prompts a strategic re-evaluation regarding how PROMs are funded, implemented, and utilized. As the first economic evaluation of PROMs in clinical practice, this study is a novel contribution to the emergent VBHC and PROMs evidence base. Furthermore, the findings from this study will inform recommendations to improve PROMs delivery across Wales.

**Supplementary Information:**

The online version contains supplementary material available at 10.1186/s41687-025-00853-w.

## Highlights

This study uses Social-Return on Investment (SROI) analysis to explore the value of Patient-Reported Outcome Measures (PROMs) within a Value-Based Healthcare (VBHC) framework. Results varied across services: Heart Failure had the highest return (5.55:1), Parkinson’s a low return (1.29:1), and Epilepsy a negative return (0.85:1). Findings indicate that PROMs generate the most social value with targeted implementation and robust infrastructure, allowing PROMs to be utilised as an effective tool for improving patient care.

**Supplementary Information:**

The online version contains supplementary material available at 10.1186/s41687-025-00853-w.

## Introduction

Value-Based Healthcare (VBHC) is a healthcare delivery model with the overarching goal of maximising value for patients and healthcare providers [1–4]. Various definitions of VBHC exist [2, 4], and within the context of the UK National Health Service (NHS), it is specifically defined as “the equitable, sustainable and transparent use of available resources to achieve better outcomes and experiences for every person” [5]. The concept of the ‘value’ in healthcare systems is gaining international prominence, driven by growing demand for services that surpass available resources [5–9]. This trend is anticipated to persist due to evolving population demographics and increasingly complex healthcare needs [10–12].

VBHC models prioritise measuring care through patient outcomes over service volume [12, 13]. As such, there is growing interest in the use of Patient Reported Outcome Measures (PROMs) within VBHC settings [12, 14]. PROMs are questionnaires which seek to comprehensively capture patient outcomes [15]. Such outcomes can include disease symptoms, mental and social functioning, and health-related quality of life [15, 16]. There are various potential uses of PROMs within a VBHC setting, including symptom monitoring, tailoring of treatment pathways, personalised care, shared decision making, and healthcare monitoring, decision making and design [17]. However, it is also important to note that VBHC is not exclusively focused or reliant on PROMs, and the primary aim of VBHC is to identify and prioritise the most relevant outcomes for specific patient populations [1, 2]. However, PROMs are increasingly adopted within VBHC frameworks, and this study focuses specifically on the role of PROMs within VBHC.

The current evidence-base regarding the effectiveness of PROMs for improving patient and service outcomes is limited. With the exception of a few well-designed studies demonstrating the potential of PROMs to improve patient outcomes in cancer patients (such as improvements in health-related quality of life and life expectancy) [17–20], robust evidence regarding the efficacy of PROMs across varying contexts is limited. Furthermore, the potential cost-effectiveness of PROM interventions remains unclear. Some studies demonstrate that PROM interventions can reduce resource utilisation through reduced patient hospitalisations and emergency visits [17–19]. However, not all studies show a reduction in healthcare demand when comparing patients receiving PROMs to a control group [17, 20]. Further research is important to understand if PROMs implemented into routine care are effective in providing the intended benefits to patients and healthcare providers.

The purpose of this study was to conduct a Social Return on Investment (SROI) analysis of PROMs implemented as part of VBHC programmes. SROIs are a methodological framework for measuring and financially quantifying the social, economic, and environmental value generated by an intervention, policy, or organization [21]. SROIs allow the calculation of a benefit-to-cost ratio that represents multiple stakeholder perspectives and captures both positive and negative outcomes [21–23]. By adopting a broader concept of value, SROIs provide a more holistic evaluation, capturing outcomes not typically measured in traditional economic methodologies (e.g., cost-effectiveness analyses) [21]. In Value-Based Healthcare (VBHC), the conceptualization of care places patients at the centre, emphasizing outcomes that matter most to patients [5, 12, 24]. This underscores the rationale for employing a SROI approach, which aligns with the patient centred ethos of VBHC. Additionally, SROIs are increasingly being used in public health and health care settings [22, 23].

### Aims and objectives

By utilising the SROI framework, this study aimed to quantify the social and economic value of PROMs implemented within a VBHC setting. This consisted of separate evaluative SROI analyses of three services within a UK health board that have adopted PROMs as part of a VBHC programme: (1) a Heart Failure Service, (2) an Epilepsy Service, and (3) a Parkinson’s Disease service. These three conditions were purposively selected to explore the universal applicability of PROMs across diverse healthcare and demographic contexts and assess their ability to bring about anticipated outcomes. We also planned to evaluate a cataract surgery service, but this was halted due to PROMs being discontinued within this service in 2021.

To the best of our knowledge, this was the first Social Return on Investment evaluation of PROMs. It is hoped that this will inform wider research on PROMs within a VBHC context and inform strategic decision-making and resource planning. The challenges of conducting SROI evaluations within a public health context were also reported.

### Ethical approval

Ethical approval for this study was granted by the Wales Research Ethics Committee 5 (ref 22/WA/0044) on 22nd March 2022. The protocol for this study was published in 2023 [25].

### Methods

The SROI methodology was implemented as part of a broader mixed methods study that incorporated a realist evaluation [26, 27], a scoping review [17], and data collection using patient and clinician questionnaires, as well as routinely collected data. We used a SROI analysis framework following the guidelines developed by Nicholls et al. [28].

### Setting

In 2015, a UK NHS Health Board, the Aneurin Bevan University Health board, undertook the integration of PROMs into routine care as part of a wider initiative to adopt VBHC [12]. This was driven by the need to address escalating healthcare costs and demand, whilst balancing the objectives of cost-effectiveness and sustained healthcare quality. The aim was to improve service efficiency and patient care within currently available resources. This was to be accomplished by tailoring treatments to individual patient symptoms and needs, with the ultimate goal of enhancing health outcomes and managing demand more effectively. Further details regarding the services and intervention are provided in Appendix 1.

### Stage 1: Establishing scope

The first stage involved establishing the scope of the analysis and identifying relevant stakeholders. We excluded outcomes considered outside the scope of this research project (Appendix 2) and restricted the analysis to a one-year timeframe. This was influenced by the practicalities of accurately measuring outcomes, and this decision ensured that our evaluation was based on robust evidence. For instance, the scarcity of routinely collected longitudinal data made it impractical to establish reliable causal relationships beyond a one-year period (Appendix 3, Table A1).

Stakeholders are groups of people or organisations that the PROMs intervention might impact. Within the scope of our evaluation, two key stakeholders were identified: (1) patients completing PROMs, and (2) the healthcare services.

### Stage 2: Mapping outcomes

Mapping outcomes involved identifying the potential changes that occur as a result of the intervention and their causal pathways. The identification of outcomes was accomplished through a scoping review and a mixed-methods realist analysis, both conducted as separate workstreams, with patient and public as well as stakeholder involvement [17, 26, 27]. The realist evaluation, which included 105 interviews with patients, caregivers, and clinicians, sought to determine what worked about PROMs, for whom, and in what circumstances. Meanwhile, the scoping review analysed 43 studies to investigate current evidence on the implementation and effectiveness of PROMs within a clinical context. From this, a theory of change was developed for each service to describe the process by which inputs led to various outcomes for each stakeholder group. The theory of change was adapted for the SROI based on the scope of the evaluation (See Appendix 2). It is important to acknowledge that VBHC definitions can differ across different healthcare systems, which will impact the theory of change. For example, in privatised healthcare systems, VBHC is primarily a tool to drive efficiency, whereas in the UK healthcare system, VBHC is primarily intended to optimise patient outcomes.

### Stage 3: Evidencing and valuing outcomes and inputs

#### Evidencing outcomes

We utilised a combination of patient and clinician questionnaire data, and longitudinal routinely collected data. Where possible, we prioritised using routine data to evidence outcomes and supplemented with questionnaire data where required. A benefit of using routinely collected data is that it incorporated a larger cohort of patients, which provides more objective data on outcomes over time. Appendix 3 (Table A1) outlines the initial outcomes we aimed to assess, the types of evidence we intended to gather, the evidence source used (routine or questionnaire data), and any outcomes that were omitted due to insufficient evidence.

The theory of change informed the outcomes measured within the questionnaires. Six separate questionnaires were developed for both patients and clinicians across the three services, and all questionnaires were translated into Welsh (see Appendix 4). Input from Patient and Public Involvement (PPI) groups was incorporated into the design of each questionnaire and information sheet (Appendix 5). The questionnaires were developed using SurveyMonkey, and were distributed by text message to eligible patients, or via email to staff [29]. A follow-up reminder was sent to patients after one week. In exchange for participation, patients were provided the option to receive a £5 Amazon voucher. All questionnaires were anonymised.

Inclusion criteria for patients included (1) being a verified patient in the Heart Failure, Epilepsy, or Parkinson’s services, and (2) being verified as having completed a PROM in the past two years as part of their care. The inclusion criteria for clinicians included that they (1) work in the Heart Failure, Epilepsy, or Parkinson’s services, and (2) have experience using PROMs clinically. Data was entered into Excel and analysed in RStudio [30, 31].

#### Measuring change

The number of patients completing PROMs between January 1st 2022 and December 31st 2022 was used as the proportion eligible to benefit from PROMs within each service. The proportion of these patients who experienced meaningful change in each outcome was determined using the questionnaire or routine data.

For the questionnaire data, material change was defined as a score of *≥* 4 on a 5-point Likert scale (i.e., ‘often’ / ‘all of the time’). The proportion of participants who answered *≥* 4 for each outcome was multiplied by the number of PROMs completers within the 2022 annual period. For routine data, we conducted case-by-case analyses to evaluate changes. See Appendix 6, Table A2, for more details on how meaningful change was calculated.

#### Valuing inputs and outcomes

The Health Board provided the costing inputs required for the delivery of the PROMs programme for the 1-year period from 1st October 2022 to 1st October 2023, reflecting cost prices as of 2023. Given that PROMs are implemented across the Health Board, total running costs were divided between all PROM using services. Such costs included staffing and software licenses. Additionally, we included the costs of text messages for requesting patients to complete PROMs sent by each service, and the labour costs for clinicians to review PROMs. Labour costs were estimated based on the annual number of completed PROMs in each service, assuming a review time of two minutes for heart failure and one minute for epilepsy and Parkinson’s, at a Band 7 nurse’s hourly rate of £64.32 [32]. These labour estimates were informed by service pathway knowledge, stakeholder consultations, and interviews from the realist analysis.

A range of sources were used to assign financial proxies to the outcomes. The main source was the Housing Associations’ Charitable Trust (HACT) Social Value Calculator version 4 [33]. National Unit Costs of Health and Social Care (2022) were used to measure staffing costs [32]. To avoid overclaiming and to accurately capture what can be reasonably expected to be achieved from PROMs, we used 10% of the financial proxy value for the majority of outcomes (See Table 4). This percentage was based on consultation with stakeholders.

### Stage 4: Establishing impact

To reduce the risk of overclaiming it was essential to account for attribution, deadweight, displacement, and drop-off. Any estimations and assumptions were determined through evidence obtained from the realist analysis and in consultation with clinical stakeholders. Assumptions used in the analysis are outlined in Table 1.

**Table 1:** Key assumptions underpinning the SROI analysis

Assumption 1:	In conducting the SROI analysis over a one-year period, we assumed that all benefits from PROMs would stop after the analysis period.
Assumption 2:	In establishing the scope of our analysis, we excluded two stakeholders: (1) family members/carers of patients, and (2) the Welsh Government as the funder of the Health Board. It was deemed that outcomes experienced by the Welsh Government would fall beyond the time period of the evaluation, and that it was beyond the remit of this analysis to capture outcomes from family members/carers. This analysis therefore assumes that only the included stakeholders benefit from PROMs.
Assumption 3:	In estimating the effect size for outcomes measured by the questionnaire, we assumed an average improvement of 10% for patients who experienced benefits from PROMs. For example, a 10% improvement in health. Therefore, in our analysis, we used 10% of the financial proxy value to account for our estimated effect size of 10%. This assumption was developed in collaboration with clinical stakeholders.
Assumption 4:	We measured attribution using questionnaire items answered only by the subset of patients who remembered completing PROMs. This was because, although all contacted patients would have completed a PROM, not all would remember this. Therefore, in our analysis, we assumed that patients who do not remember completing a PROM questionnaire benefit to the same extent as patients who do remember completing a PROM questionnaire.
Assumption 5:	For our questionnaire data analysis, we estimated a threshold at which we assumed ‘meaningful change’ for an outcome was achieved. This threshold was determined as *≥* 4 on a 5-point Likert scale. This assumption applied to the questionnaire data analysis for estimating both impact and attrition.
Assumption 6:	For estimating attribution and displacement, we made assumptions in collaboration with stakeholders and through careful analysis of service and qualitative data. Table S2-S3 provide a thorough breakdown of each assumption.

#### Attribution

Attribution refers to the degree of change that stakeholders experience from other variables separate to the PROMs intervention. We utilised questionnaire items to assess the attribution rates for each outcome. For instance, one of the questions aimed at determining attribution was, “PROMs have helped me to monitor my heart failure symptoms.” We determined the attribution from the proportion of patients who reported meaningful change due to PROMs. The threshold for meaningful change was set as > 4 on a 5-point Likert scale (i.e., ‘large amount’ / ‘very large amount’). To see how attribution was determined for each outcome in more detail, see Appendix 6, Table A3.

#### Deadweight

Deadweight refers to the proportion of change that would have occurred if PROMs had never been implemented in the health service. Deadweight was calculated using the HACT recommendations for each outcome (Table 4).

#### Displacement

Displacement refers to the potential outcomes that are being displaced by PROMs; for example, a reduction in waiting lists for one service could be due to patient referrals, thus increasing waiting lists in a different service. Displacement is ideally determined using objective data. However, due to the lack of data to inform displacement, we consulted process maps, qualitative interviews, and clinical stakeholders. The methods and assumptions used to determine the estimates for displacement are detailed in Appendix 6, Table A4.

#### Drop-off

Drop-off refers to the proportion of an outcome that will diminish each year. As we conducted an evaluation for a one-year period, we assumed that 100% of outcomes would stop after the analysis period.

## Results

### Patient questionnaire

Across the three conditions, 230 participants were recruited for the patient questionnaires, and 14 participants were recruited for the staff questionnaires (Table 2).

**Table 2:** Participant recruitment for patient and clinician questionnaires

	Number of Participants Contacted	Number who completed the questionnaire	Response Rate	Participants who remember completing a PROM
**Patient Questionnaire**				
Epilepsy	884	66	7.5%	32 (48%)
Heart Failure	2,388	140	5.9%	31 (22%)
Parkinson’s	305	24	7.9%	10 (42%)
Total	3,577	230	6.4%	73 (32%)
**Clinician Questionnaire**				
Epilepsy	-	5	-	-
Heart Failure	-	6	-	-
Parkinson’s	-	3	-	-
Total	77	14	18.2%	-

The percentage of participants who remembered completing PROMs was calculated from the corresponding sample size of survey participants in each service

Overall, the demographic characteristics were well-balanced between patients who reported remembering PROMs, and those who did not (Table 3). Additionally, the demographic characteristics between PROM completing patients across the whole service and the online questionnaire sample was well-balanced (Table 2). Questionnaire results are presented in detail in Appendix 7.

**Table 3:** Baseline demographic characteristics for heart failure, Epilepsy, and Parkinson’s patients

Demographic Characteristics of Heart Failure Patients			
	All Patients in the Heart Failure Services who completed at least one PROM in 2022 (*n* = 1113)	Heart Failure Patients from Online Survey (*n* = 140)	Heart Failure Patients from Online Survey who remember completing a PROMs (*n* = 32)
**Age (years)**			
18–35	19 (2%)	2 (1%)	2 (6%)
36–50	77 (7%)	8 (6%)	2 (6%)
51–70	395 (35.6%)	55 (40%)	16 (50%)
71+	620 (55.8%)	74 (53%)	12 (38%)
Missing	2	-	-
**Gender**			
Female	355 (32%)	50 (36%)	15 (47%)
Male	756 (68%)	89 (64%)	17 (53%)
Other	2	1 (2%)	0 (0%)
**Ethnicity**			
Asian or Asian British	2 (1%)	1 (3%)	9 (1%)
Black, Black British, Caribbean, or African	2 (1%)	1 (3%)	2 (< 1%)
Mixed or multiple ethnic group	1 (< 1%)	1 (3%)	-
White	132 (95%)	28 (88%)	862 (98.6%)
Other	2 (1%)	1 (3%)	1 (< 1%)
Missing	-	-	239

Demographic Characteristics of Epilepsy Patients			
	All Patients in the Epilepsy Services who completed at least one PROM in 2022 (*n* = 304)	Epilepsy Patients from Online Survey (*n* = 66)	Epilepsy Patients from Online Survey who remember completing a PROMs (*n* = 31)
**Age (years)**			
Under 18	6 (2%)	-	-
18–35	141 (47%)	30 (45%)	15 (48%)
36–50	90 (30%)	21 (32%)	11 (36%)
51–70	57 (19%)	12 (18%)	5 (16%)
71+	9 (3%)	2 (3%)	0 (0%)
Prefer not to say	-	2 (2%)	0 (0%)
Missing	1	-	-
**Gender**			
Female	211 (70%)	42 (80%)	26 (84%)
Male	92 (30%)	11 (17%)	5 (16%)
Other	-	2 (3%)	0 (0%)
Missing	1	-	-
**Ethnicity**			
Mixed or multiple ethnic group	-	2 (3%)	1 (3%)
White	-	62 (94%)	29 (94%)
Other	-	1 (2%)	1 (3%)
Prefer not to say	-	1 (2%)	0 (0%)
Missing	304	-	-

Demographic Characteristics of Parkinson’s Patients			
	All Patients in the Parkinson’s Services who completed at least one PROM in 2022 (*n* = 170)	Parkinson’s Patients from Online Survey (*n* = 24)	Parkinson’s Patients from Online Survey who remember completing a PROMs (*n* = 10)
**Age (years)**			
18–35	10 (6%)	2 (8%)	0 (0%)
36–50	99 (58%)	17 (71%)	7 (70%)
51–70	61 (36%)	5 (21%)	3 (30%)
**Gender**			
Female	61 (36%)	7 (29%)	2 (20%)
Male	109 (64%)	17 (71%)	8 (80%)
**Ethnicity**			
White	-	24 (100%)	10 (100%)
Missing	170	-	-

Data displayed as number of participants (%). Percentages are rounded up to nearest whole number. Percentages are calculated from the corresponding sample size of survey participants in each service. Missing data was excluded from percentage calculations

### Inputs

Using the high-level cost information provided by the health board, the cost of running PROMs in each service was estimated as £37,845 for Heart Failure, £15,725 for Parkinson’s Disease, and £17,077 for Epilepsy.

### Outputs, outcomes and social value

To quantify the social benefits of PROMs, data from the questionnaire was used to estimate the proportion of patients completing PROMs in each service who have benefited from PROMs. The attribution rate varied for each outcome. Attribution of social value to other processes aside from PROMs varied from 40 to 100% (Table 4). The estimated displacement varied from 0 to 10%, except for the Heart Failure outcome ‘Reduced present demand on health service’, for which we assigned a 75% displacement rate following consultation with clinical stakeholders. A deadweight of 27% was applied to all outcomes, consistent with methodologies of the HACT Social Value Calculator [33]. The questionnaire used to rank outcomes by priority was discarded due to poor data quality.

**Table 4:** Stakeholder outcomes, financial proxies and the social value generated for each stakeholder group measured over a one-year timeframe

Stakeholder	Outcome	Financial Proxy	Value of Financial Proxy	Estimated Number Experiencing the Outcome in 2022	Attribution to Other Reasons	Deadweight		Displacement	Net social Value
**Social Value Generated for Heart Failure Stakeholders**									
Heart Failure Patients who have completed PROMs in 2022 (*n* = 1113)	Improved management of patients heart failure leads to slightly better health and health outcomes.	Good Overall Health HACT, > 50, Outside of London	£2,018.6 †	924	100%	27%		10%	£0
	PROMs help patients to be more knowledgeable, and confident in managing their heart failure.	Feel in control of life HACT, > 50, Outside of London	£1,573.4 †	423	75%	27%		0%	£121,462.55
	Triage of patients improves service efficiency and leads to more rapid access to care when needed.	Average cost of private healthcare appointment in the UK (2022).	£195	378	80%	27%		10%	£14,528.24
	PROMs help patients feel more listened to and supported by their healthcare providers due to the provision of more patient-centred care.	Able to obtain advice locally HACT, > 50, Outside of London	£245.7 †	545	75%	27%		0%	£26,027.16
Heart Failure Service	Reduced present demand on health service (reduced 1 FTE caseload).	The average FTE salary for a nurse at the Heart Failure Service.	£42,332.02	10	40%	27%		75%	£46,353.56
								**TOTAL**	**£208**,**371.51**
**Social Value Generated for Epilepsy Stakeholders**									
Epilepsy patients who have completed PROMs in 2022 (*n* = 304)	Improved management of patients epilepsy leads to slightly better epilepsy-related health.	Good Overall Health HACT, 25–49 years of age, Outside of London	£2092.2 †	119	97%	27%	0%		£4,907.23
	Due to improved identification and signposting to mental health support, patients have improved mental health.	Relief from anxiety and depression, 25–49 years of age, Outside of London	£3670.6 †	12	100%	27%	10%		£0
	PROMs help patients to be more knowledgeable and confident in managing their epilepsy and mental health.	Feel in control of life, 25–49 years of age, Outside of London	£1647.4 †	119	94%	27%	0%		£8,586.58
	PROMs help patients feel more listened to and supported by their healthcare providers due to the provision of more patient-centred care.	Able to obtain advice locally HACT, 25–49, Outside of London	£245.7 †	116	94%	27%	0%		£1,248.36
							**TOTAL**		**£14**,**742.17**
**Social Value Generated for Parkinson’s Stakeholders**									
Parkinson’s patients who have completed PROMs in 2022 (*n* = 170)	PROMs help patients to be more knowledgeable and confident in managing their Parkinson’s.	Feel in control of life HACT, > 50, Outside of London	£1573.4 †	79	70%	27%		0%	£18,146.44
	PROMs help patients feel more listened to and supported by their healthcare providers due to the provision of more patient-centred care.	Able to obtain advice locally HACT, > 50, Outside of London	£245.7 †	121	90%	27%		0%	£2,152.33
								**TOTAL**	**£20**,**298.77**

† Monetary valuation used is 10% of the original financial proxy indicating an estimated 10% improvement in outcome per stakeholder that experiences change

Attribution is defined as the percentage of outcomes which are attributable to activities other than PROMs i.e., an attribution rate of 100% means that 100% of the outcome is due to other activities

Table 4 shows the number of people experiencing material changes for each outcome, and the resulting social value generated when the attribution, deadweight, and displacement were applied. In total, the social value was generated by PROMs within a one-year period was £203,777.10 for Heart Failure, £14,742.17 for Epilepsy, and £20,298.77 for Parkinson’s Disease.

### Stage 5: Calculating the social return on investment ratio

To calculate the base case SROI ratio, the total social value of benefits experienced by stakeholders was divided by the value of inputs required to deliver the PROMs program. For Heart Failure, this yielded a base case SROI ratio of £5.51 of social value generated for every £1 spent. For Epilepsy, this yielded a base case SROI ratio of £0.85 of social value generated for every £1 spent. For Parkinson’s Disease, this yielded a base case SROI ratio of £1.29 of social value generated for every £1 spent.

### Sensitivity analysis

A series of pre-determined sensitivity analyses were conducted to assess the robustness of the assumptions underlying the base case scenario (Table 5). The small range across the three SROI analyses suggests that the base case scenario is robust.

**Table 5:** Sensitivity analysis

Base Case	Revised Scenario	Revised Ratio
**Sensitivity Analysis for Heart Failure SROI**		
The outcome ‘PROMs help patients to be more knowledgeable, and confident in managing their heart failure.’ is measured using the questionnaire item ‘I have been better able to monitor my heart failure symptoms.’ The attribution for this outcome is measured using the questionnaire item ‘Because of PROMs, I have been better able to monitor my heart failure symptoms.’	The outcome ‘PROMs help patients to be more knowledgeable, and confident in managing their heart failure.’ is measured using the questionnaire item ‘I have a better understanding and awareness of heart failure and my individual symptoms’. The attribution for this outcome is measured using the questionnaire item ‘Because of PROMs, I have a better understanding and awareness of heart failure and my individual symptoms’.	6.35:1
The outcome ‘PROMs help patients feel more listened to and supported by their healthcare providers due to the provision of more patient-centred care’ is measured using the questionnaire item ‘In my heart failure care, my clinicians have listened to me more’.	The outcome ‘PROMs help patients feel more listened to and supported by their healthcare providers due to the provision of more patient-centred care’ is measured using the questionnaire item ‘I have felt more informed and supported in achieving any individual health goals I have with my heart failure’	5.27:1
Attribution to other variables for ‘Reduced present demand on health service’ measured’ estimated at 75%.	Attribution to other variables for ‘Reduced present demand on health service’ measured’ estimated at 50%.	6.73:1
Proportion of financial proxies for specific outcomes set at 10% of the original proxy value.	Proportion of financial proxies for specific outcomes set at 5% of the original proxy value.	3.56:1
Proportion of financial proxies for specific outcomes set at 10% of the original proxy value.	Proportion of financial proxies for specific outcomes set at 15% of the original proxy value.	7.45:1
**Sensitivity Analysis for Epilepsy SROI**		
The attribution for outcome ‘Due to improved identification and signposting to mental health support, patients have improved mental health’ is measured using the questionnaire item ‘Because of PROMs, there are more optimal care pathways in heart failure care based on individual health needs’.	The attribution for outcome ‘Due to improved identification and signposting to mental health support, patients have improved mental health’ is measured using the questionnaire item ‘To what extent do you think that your mental health and/or mental health care improved because of PROMs’.	0.95:1
The outcome ‘PROMs help patients to be more knowledgeable, and confident in managing their epilepsy.’ is measured using the questionnaire item ‘I have been better able to monitor my epilepsy symptoms’. The attribution for this outcome is measured using the questionnaire item ‘Because of PROMs, I have been better able to monitor my epilepsy symptoms.’	The outcome ‘PROMs help patients to be more knowledgeable, and confident in managing their epilepsy.’ is measured using the questionnaire item ‘I have a better understanding and awareness of epilepsy and my individual symptoms.’ The attribution for this outcome is measured using the questionnaire item ‘Because of PROMs, I have a better understanding and awareness of epilepsy and my individual symptoms.’	0.35:1
Proportion of financial proxies for specific outcomes set at 10% of the original proxy value.	Proportion of financial proxies for specific outcomes set at 5% of the original proxy value.	0.43:1
Proportion of financial proxies for specific outcomes set at 10% of the original proxy value.	Proportion of financial proxies for specific outcomes set at 15% of the original proxy value.	1.28:1
**Sensitivity Analysis for Parkinson’s SROI**		
The outcome ‘PROMs help patients to be more knowledgeable, and confident in managing their Parkinson’s.’ is measured using the questionnaire item ‘I have been better able to monitor my Parkinson’s symptoms.’ The attribution for this outcome is measured using the questionnaire item ‘Because of PROMs, I have been better able to monitor my Parkinson’s symptoms.’	The outcome ‘PROMs help patients to be more knowledgeable, and confident in managing their epilepsy.’ is measured using the questionnaire item ‘I have a better understanding and awareness of Parkinson’s and my individual symptoms.’ The attribution for this outcome is measured using the questionnaire item ‘Because of PROMs, I have a better understanding and awareness of Parkinson’s and my individual symptoms.’	2.48:1
The outcome ‘PROMs help patients feel more listened to and supported by their healthcare providers due to the provision of more patient-centred care’ is measured using the questionnaire item ‘In my Parkinson’s care, my clinicians have listened to me more’. The attribution for this outcome is measured using the questionnaire item ‘Because of PROMs, I have felt more listened to by my clinicians in my Parkinson’s care’.	The outcome ‘PROMs help patients feel more listened to and supported by their healthcare providers due to the provision of more patient-centred care’ is measured using the questionnaire item ‘I have felt more informed and supported in achieving any individual health goals I have with my Parkinson’s’. The attribution for this outcome is measured using the questionnaire item ‘Because of PROMs, I have felt more informed and supported in achieving any individual health goals I have with my Parkinson’s’.	1.32:1
Proportion of financial proxies for specific outcomes set at 10% of the original proxy value.	Proportion of financial proxies for specific outcomes set at 5% of the original proxy value.	0.65:1
Proportion of financial proxies for specific outcomes set at 10% of the original proxy value.	Proportion of financial proxies for specific outcomes set at 15% of the original proxy value.	1.94:1

## Discussion

Overall, findings show that the utilisation of PROMs in routine healthcare presents complexities, with wide variations in value derived across different services. Where PROMs demonstrated substantial social and economic return on investment, such as in Heart Failure, real transformation was seen within the service. However, where PROMs provided low value, such as in Epilepsy, few of the anticipated patient benefits were achieved, raising questions about the efficacy and justification of allocating resources towards the collection of PROMs in their current configuration. These findings demonstrate the nuanced nature of PROMs implemented within a VBHC framework, and careful consideration is likely needed in selecting suitable services and tailoring the implementation of PROMs to meet specific service and patient requirements effectively. As the SROI of PROMs implemented within a healthcare setting, this study provides a valuable addition to the VBHC and PROMs evidence base.

In our analysis, PROMs provided the most social value within Heart Failure Services with a base case SROI ratio of 5.55:1 (3.56–7.45). The results from the Heart Failure Service demonstrate the potential of PROMs to improve patient and healthcare service outcomes, and these findings add to emerging evidence demonstrating the benefits of PROM interventions [16, 17]. However, our analysis also revealed that PROMs had a small return of investment in Parkinson’s services with a ratio of 1.29:1 (0.65–2.48), and no return on investment in Epilepsy services with an SROI ratio of 0.85:1 (0.43–1.28).

One purpose of an SROI analysis is to identify areas for improvement in the structure, implementation, or resource allocation of the intervention. Our analysis revealed that the implementation of PROMs did not occur as envisaged in the theory of change. However, a less favourable SROI ratio, as seen in Epilepsy and Parkinson’s Disease services, does not necessarily lead to the conclusion that the PROMs intervention is of no benefit. These findings suggest that there may be aspects of the program design or resource allocation that are not fully optimized, thereby limiting the value that could be achieved for patients and services. Furthermore, variations in the value of PROMs between services may also partly be explained by the theory that PROMs are more effective in some conditions and patient populations [24].

An additional consideration for the future implementation and refinement of PROMs is to improve the SROI ratio by reducing the intervention costs per patient. Potential options for cost reduction could include improving IT efficiencies, exploring more affordable methods for distributing PROMs beyond text messaging, and utilising bulk licensing or shared infrastructure. By adopting cost-reduction strategies that do not compromise patient care quality, the SROI of PROMs could be improved.

From our questionnaires, only 22 − 48% of participants recalled PROMs, showing that majority of patients struggled to remember the PROMs they had completed (Table 2). This raises important considerations concerning the value patients can be expected to gain from a tool they don’t remember or understand. An important question for future research is whether increased patient awareness and engagement would lead to more value generated across patient-mediated outcomes. Together, our findings show that there is a gap between the intended purposes of PROMs and the actual patient experience.

While the SROI provided a quantitative measure of value, a parallel Realist Evaluation provided a deeper qualitative perspective, offering further insights into why Heart Failure derived more value from PROMs [26, 27]. The incorporation of a Realist Evaluation alongside the SROI enabled a more comprehensive evaluation of PROMs. As the PROMs programme was fully integrated into the services, there was a challenge in separating the impact and mechanisms of the PROMs intervention from the rest of the service. The realist approach was instrumental in unravelling these factors.

The Realist Evaluation highlighted factors that led to the success of PROMs in Heart Failure services. For instance, the Heart Failure service used PROMs scores to develop a tailored care pathway which led to more efficient triage, prompt diagnosis, more efficient referrals, reduction in readmissions, and reduced waiting times [34]. In contrast, the analysis showed that a less customised implementation of PROMs in Parkinson’s disease restricted the value generated by failing to target the specific needs of the service and patients. These findings are consistent with VBHC theories that a comprehensive and tailored approach is required for optimal results [24, 35]. The Realist Evaluation also identified factors creating barriers across all services, such as lack of IT integration with patient records and resource constraints, which limited the integration of PROMs into routine care. For example, a lack of mental health resources was a significant barrier to achieving the aim of improving mental health treatment for Epilepsy patients, a finding strongly reflected in the SROI analysis. Together, the SROI and Realist evaluation highlight the importance of considering systemic challenges that might hinder the social and economic value of PROMs.

The varying SROI ratios of the PROM intervention across the three service suggests a need for a strategic re-evaluation regarding how PROMs should be implemented in routine care. Specifically, the underwhelming results observed in the epilepsy services raise important questions about how to interpret and act upon these findings. The findings from this study will be used by policy and clinical leads to inform the future delivery of PROMs at a national level. Through discussing the findings of this analysis with senior clinical and policy stakeholders in Wales, it was decided that the current implementation of PROMs needs careful revision with the aim of more efficient resource allocation and improved patient outcomes. Internationally, the current evidence-base for VBHC and PROMs is too limited to draw conclusions about the value of PROM interventions. Additionally, for health services interested in implementing PROMs into routine care, our findings demonstrate the variability and uncertainty in predicting implementation success. For VBHC and PROMs to gain leverage and credibility, more research is needed that demonstrates value for patients and healthcare providers.

### Strengths

There are key strengths to our study. This was a rigorous analysis for which we adhered to the SROI protocol as developed by Nicholls et al. [28]. We provide full transparency through documentation of each step in our evaluation (See Appendix). We performed sensitivity analyses to further test the robustness of the underlying assumptions. Additionally, the demographic characteristics of the patient questionnaire sample were largely representative of the patient population.

### Limitations

The findings of this analysis should be viewed in the context of the following limitations. This SROI was analysed over a one-year period and may not accurately capture the long-term value and cost-saving potential of VBHC and PROMs interventions. The patient questionnaire for the Parkinson’s SROI and all staff questionnaires obtained small sample sizes. Due to practical constraints, this SROI evaluation relied on retrospective, non-validated questionnaires instead of a before-and-after or case-control design [23]. This limited our ability to precisely measure outcome changes and assumptions were required to account for this. The retrospective data collection in our study introduces the risk of recall bias, and our reliance on a subset of patients who remembered completing PROMs increases the risk of selection bias in our attribution estimates. Certain outcomes were excluded from the scope of the analysis due to the challenges in adequately measuring impact and attribution. The exclusion of outcomes was mostly due to a lack of routine or longitudinal data, and longer-term outcomes were most affected (see Appendix 3, Table A1).

### Challenges & lessons

Conducting SROI analyses in healthcare present distinct challenges [36]. As reflected in other studies, service level data is frequently scarce, and there are often limited resources within healthcare settings to extract administrative data for research purposes [36]. In this study, this led to a wide variation in the number of measurable outcomes across the three services. For instance, we measured five outcomes for Heart Failure compared to two for Parkinson’s Disease. This illustrated that despite SROIs aims to fully capture value generated, this comprehensiveness is often not achieved. Furthermore, in conducting three separate analyses, our study highlights the variation in quality and reliability that can emerge despite a consistent application of SROI methodology.

The challenges of securing high-quality routine data for SROI analyses impacts the reliability and robustness of findings. The ideal method of a comparator group for determining attribution and deadweight is often resource-intensive and, as in the case of this study, impractical [21, 23, 36]. This challenge was exacerbated in the evaluation of a complex intervention such as PROMs, which was deeply embedded within the service pathway. Future research should prioritize acquiring longitudinal data, and VBHC initiatives should consistently gather data on key service and patient outcomes to enable a data-driven evaluations.

## Conclusions

This study is the first rigorously conducted SROI analysis of a PROMS intervention implemented in a VBHC context, revealing varied impacts across Heart Failure, Parkinson’s, and Epilepsy services. While Heart Failure Services demonstrated substantial value, illustrating that when utilised appropriately PROMs can yield high return on investment, the limited effectiveness of PROMs in Parkinson’s and Epilepsy services demonstrates that the impact of PROMs is not universal in all contexts. Potential explanations include sub-optimal programme design, systemic barriers to implementation, inefficient resource allocation, or that PROM interventions are not universally effective for all populations/services. These findings emphasise that how PROMs are implemented is likely crucial for realising a high return on investment. As the evidence base for VBHC and PROMs is in its infancy [37], ongoing research is essential to inform evidenced-based healthcare decisions in the implementation and delivery of these programs.

## Electronic supplementary material

Below is the link to the electronic supplementary material.

Supplementary Material 1

## Data Availability

Data available upon request.

## Abbreviations

SROI: Social-Return on Investment
VBHC: Value Based Healthcare
PROMs: Patient Reported Outcome Measures
HACT: Housing Associations’ Charitable Trust

## References

[1] Teisberg E, Wallace S, O’Hara S (2020) Defining and implementing value-based Health Care: a Strategic Framework. Acad Med 95(5). 10.1097/ACM.0000000000003122 10.1097/ACM.0000000000003122 PMC7185050 31833857

[2] Porter ME (2010) What is value in health care? N Engl J Med 363(26). 10.1056/nejmp1011024 10.1056/NEJMp1011024 21142528

[3] Putera I (2017) Redefining Health: implication for value-based Healthcare Reform. Cureus Published Online. 10.7759/cureus.1067 10.7759/cureus.1067 PMC5376155 28409068

[4] Gray M (2017) Value based healthcare. BMJ 356. 10.1136/BMJ.J437 10.1136/bmj.j437 28130219

[5] Hurst L, Mahtani K, Pluddemann A et al Defining value-based Healthcare in the NHS. Centre Evidence-Based Med Rep 2019/04. 2019;(4).

[6] Kaplan RS, Porter ME (2011) How to solve the cost crisis in health care. Harv Bus Rev 89(9) 21939127

[7] Browne JP, Cano SJ, Smith S (2017) Using patient-reported outcome measures to improve health care: time for a new approach. Med Care 55(10). 10.1097/MLR.0000000000000792 10.1097/MLR.0000000000000792 28857961

[8] Peters M, Crocker H, Jenkinson C, Doll H, Fitzpatrick R (2014) The routine collection of patient-reported outcome measures (PROMs) for long-term conditions in primary care: a cohort survey. BMJ Open 4(2). 10.1136/BMJOPEN-2013-003968 10.1136/bmjopen-2013-003968 PMC3931992 24561495

[9] Herzlinger RE (2011) How to solve the cost crisis in health care. Harv Bus Rev. 89(11) 22111427

[10] McKee M, Dunnell K, Anderson M et al (2021) The changing health needs of the UK population. Lancet 397(10288):1979. 10.1016/S0140-6736(21)00229-4 33965065 10.1016/S0140-6736(21)00229-4 PMC9751760

[11] Gentry S, Badrinath P (2017) Defining Health in the era of Value-based Care: lessons from England of Relevance to Other Health systems. Cureus Published Online. 10.7759/cureus.1079 10.7759/cureus.1079 PMC5383371 28405529

[12] Withers K, Palmer R, Lewis S, Carolan-Rees G (2021) First steps in PROMs and PREMs collection in Wales as part of the prudent and value-based healthcare agenda. Qual Life Res 30(11):3157–3170. 10.1007/S11136-020-02711-2 33249539 10.1007/s11136-020-02711-2 PMC7700742

[13] Eijsink JFH, Fabian AM, Vervoort JPM, Al Khayat MNMT, Boersma C, Postma MJ (2023) Value-based health care in western countries: a scoping review on the implementation of patient-reported-outcomes sets for hospital-based interventions. Expert Rev Pharmacoecon Outcomes Res 23(1). 10.1080/14737167.2023.2136168 10.1080/14737167.2023.2136168 36300427

[14] Squitieri L, Bozic KJ, Pusic AL (2017) The role of patient-reported outcome measures in Value-based payment reform. Value Health 20(6):834. 10.1016/J.JVAL.2017.02.003 28577702 10.1016/j.jval.2017.02.003 PMC5735998

[15] Porter I, Gonçalves-Bradley D, Ricci-Cabello I et al (2016) Framework and guidance for implementing patient-reported outcomes in clinical practice: evidence, challenges and opportunities. J Comp Eff Res 5(5). 10.2217/cer-2015-0014 10.2217/cer-2015-0014 27427277

[16] Gibbons C, Porter I, Gonçalves-Bradley DC et al (2021) Routine provision of feedback from patient-reported outcome measurements to healthcare providers and patients in clinical practice. Cochrane Database Syst Reviews 2021(10). 10.1002/14651858.CD011589.pub2 10.1002/14651858.CD011589.pub2 PMC8509115 34637526

[17] Silveira Bianchim M, Crane E, Jones A et al (2023) The implementation, use and impact of patient reported outcome measures in value-based healthcare programmes: a scoping review. PLoS ONE 18(12):e0290976. 10.1371/JOURNAL.PONE.0290976 38055759 10.1371/journal.pone.0290976 PMC10699630

[18] Demedts I, Himpe U, Bossuyt J et al (2021) Clinical implementation of value based healthcare: impact on outcomes for lung cancer patients. Lung Cancer 162:90–95. 10.1016/J.LUNGCAN.2021.10.010 34763159 10.1016/j.lungcan.2021.10.010

[19] Basch E, Deal AM, Kris MG et al (2016) Symptom Monitoring with patient-reported outcomes during Routine Cancer treatment: a Randomized Controlled Trial. J Clin Oncol 34(6):557–565. 10.1200/JCO.2015.63.0830 26644527 10.1200/JCO.2015.63.0830 PMC4872028

[20] Wheelock AE, Bock MA, Martin EL et al (2015) SIS.NET: a randomized controlled trial evaluating a web-based system for symptom management after treatment of breast cancer. Cancer 121(6):893–899. 10.1002/CNCR.29088 25469673 10.1002/cncr.29088

[21] Hutchinson CL, Berndt A, Forsythe D, Gilbert-Hunt S, George S, Ratcliffe J (2019) Valuing the impact of health and social care programs using social return on investment analysis: how have academics advanced the methodology? A systematic review. BMJ Open 9(8). 10.1136/bmjopen-2019-029789 10.1136/bmjopen-2019-029789 PMC6720245 31446413

[22] Laing CM, Moules NJ (2017) Social Return on Investment: a New Approach to understanding and advocating for value in Healthcare. J Nurs Adm 47(12):623–628. 10.1097/NNA.0000000000000557 29135853 10.1097/NNA.0000000000000557

[23] Banke-Thomas AO, Madaj B, Charles A, Van Den Broek N (2015) Social Return on Investment (SROI) methodology to account for value for money of public health interventions: a systematic review. BMC Public Health 15(1). 10.1186/s12889-015-1935-7 10.1186/s12889-015-1935-7 PMC4477315 26099274

[24] Lewis S (2022) Value-based healthcare: is it the way forward? Future Healthc J 9(3). 10.7861/fhj.2022-0099 10.7861/fhj.2022-0099 PMC9761467 36561818

[25] Roberts G, Cahill A, Lawthom C et al (2023) Protocol for a realist and social return on investment evaluation of the use of patient-reported outcomes in four value-based healthcare programmes. BMJ Open 13(4):e072234. 10.1136/BMJOPEN-2023-072234 37105686 10.1136/bmjopen-2023-072234 PMC10151973

[26] Bianchim MS, McLaughlin L, Roberts G, et al (2024) The use of patient-reported outcomes in acute and long-term care value-based healthcare programmes: A realist evaluation 10.1111/jan.70018 PMC12994664 40726099

[27] Bianchim MS, Roberts G, Mclaughlin L et al (2024) The Use of Patient-Reported Outcomes in Neurology Value-Based Healthcare: A Realist Evaluation

[28] Jeremy Nicholls, Lawlor E, Neitzert E et al Social Return on Investment: A guide to Social Return on Investment. Update. 2012;(January).

[29] SurveyMonkey Inc www.surveymonkey.com

[30] RStudio Team. RStudio: Integrated Development for R. RStudio, Inc., Boston, MA; 2021

[31] Microsoft, Excel [Internet]. Microsoft Corporation

[32] Jones K, Weatherly HLA, Birch S et al Unit costs of Health and Social Care 2022. Published online 2022. 10.22024/UNIKENT/01.02.100519

[33] HACT S (2023) Community investment and homelessness values from the social value bank. Accessed November 27. https://hact.org.uk/

[34] Improving outcomes for patients with heart failure with reduced ejection fraction. Life Sciences. Accessed December 14, (2023). https://lshubwales.com/success-stories/heart-failure

[35] Teisberg E, Wallace S, O’Hara S (2020) Defining and implementing value-based Health Care. Acad Med 95(5). 10.1097/acm.0000000000003122 10.1097/ACM.0000000000003122 PMC7185050 31833857

[36] Arvidson M, Lyon F, McKay S, Moro D (2013) Valuing the social? The nature and controversies of measuring social return on investment (SROI). Voluntary Sect Rev 4(1). 10.1332/204080513x661554

[37] Walraven J, Jacobs MS, Uyl-de Groot CA (2021) Leveraging the similarities between cost-effectiveness analysis and value-based Healthcare. Value Health 24(7). 10.1016/j.jval.2021.01.010 10.1016/j.jval.2021.01.010 34243828

