# Supplementary material for: A social return on investment analysis of patient-reported outcome measures in value-based healthcare
Source: J Patient Rep Outcomes. 2025 Feb 20;9:22. doi: 10.1186/s41687-025-00853-w (PMC11842646; doi:10.1186/s41687-025-00853-w)
Supplement: Supplementary file 1 — Supplementary Material 1 [file 41687_2025_853_MOESM1_ESM.docx]

**Appendix**

**A Social Return on Investment Analysis of Patient-Reported Outcome Measures in Value-Based Healthcare**

**Appendix 1: Service and PROMs intervention structures2**

Epilepsy Service2

Heart Failure Service2

Parkinson’s Service3

Process Maps for the PROMs Intervention. 4

**Appendix 2: Theory of Change Models7**

Theory of Change for PROMs within Epilepsy7

Theory of Change for PROMs within Heart Failure8

Theory of Change for PROMs within Parkinson’s9

**Appendix 3: Obtaining data for the SROI analyses10**

Table A1. Data we sought to obtain for the SROI analysis, availability of data, and outcome. 10

**Appendix 4: Questionnaire13**

Participant information sheet for patient questionnaires 13

Consent form for patient and staff questionnaires17

Questionnaire Structure for patient and staff questionnaires18

Questionnaire for Epilepsy Patients18

Questionnaire for Heart Failure Patients28

Questionnaire for Parkinson’s Patients39

Participant information sheet for staff questionnaires50

Questionnaire for Clinical Staff in the Epilepsy Service53

Questionnaire for Clinical Staff in the Heart Failure Service58

Questionnaire for Clinical Staff in the Parkinson’s Service63

**Appendix 5: Patient and Public Involvement68**

**Appendix 6: Calculating Impact69**

Table A2. Calculating meaningful change for each outcome69

Table A3. Measuring attribution for each outcome73

Table A4. Measuring displacement for each outcome77

**Appendix 7: Questionnaire Results80**

Epilepsy patient and staff questionnaire80

Heart failure patient and staff questionnaire83

Parkinson’s patient and staff questionnaire86

**Appendix 1:** PROMs service and intervention structure for the Heart Failure, Epilepsy, and Parkinson’s Services

**Epilepsy**

Epilepsy

The Adult Epilepsy Service at ABUHB caters to a population exceeding 6,000 individuals with epilepsy, offering an open access system for timely and responsive care for this unpredictable condition. In 2018/2019, Value-Based Healthcare (VBHC) principles were integrated into the Epilepsy service, aiming to utilize Patient-Reported Outcome Measures (PROMs) for a comprehensive understanding and quantification of anxiety and depression in epilepsy patients. The primary focus was to enhance mental health management at an individual patient level. Incorporating PROMs into routine care sought a data-driven assessment of outcomes and costs, aiming to improve efficiency, effectiveness, and the correlation between patient mood, medication, and treatment options. The Epilepsy PROM encompasses inquiries on seizure frequency, mood scores, and a patient's global impression of change.

*Intervention:*

The current care pathway involves new patients receiving a baseline PROM at their initial engagement with services. Existing patients in the system undergo PROMs in two distinct pathways:

1. Ad hoc, where staff identifies a need to send PROMs based on concerns about mental health.
2. A PROMs coordinator sends PROMs to patients before scheduled appointments.

Following this, the PROMs coordinator evaluates all PROMs scores, leading to various outcomes:

1. No action if no changes in mood are observed, with patients receiving a letter confirming no identified concerns and a continuation of PROMs.
2. Patients with mild/moderate mood disorders receive a letter directing them to resources and providing instructions for self-referral to online Cognitive Behavioural Therapy (CBT) through Silver Cloud.
3. Patients with severe mood disorders are referred to the community mental health team (CMHT).

**Heart Failure**

Patient Reported Outcome Measures (PROMs) were introduced into the ABUHB service in 2018. Heart Failure is a chronic and deteriorating condition and although it cannot usually be cured, the symptoms can be controlled for many years. The Heart Failure service is a nurse-led team within ABUHB and provides care primarily for patients diagnosed with Heart Failure with Reduced Ejection Fraction (HFrEF). In 2018, these patients compromise 11.3% of the total population in the health board.

PROMs were introduced into the heart failure, as the service was overwhelmed with patients and was unable to meet demand. Patients were facing long-waiting times for appointments for services which were sometimes unrelated to their symptoms. PROMs were implemented in the service in 2018/2019 with the main priorities of ensuring that those with more urgent needs could access the service rapidly to prevent deterioration and avoidable hospital admissions; and to ensure timely optimisation of evidenced-based treatment to prevent mortality and morbidity.

**Intervention**

Since the initial implementation, the care pathway has undergone several evolutions. Patients access the Heart Failure service through Electronic Referral (from GP/Cardiologist) or via the inpatient service (Cardiology Wards). A block booking system streamlines appointment scheduling for medication optimization. New patients undergo baseline PROMs [P1] and Clinical Reported Outcome Measures 1 (CROMs) [C1] during an initial nurse-led phone consultation (PROMs can be done via phone or on Dr Doctor). This information tailors subsequent appointments, with shorter 10-minute clinics for less symptomatic patients [i.e., optimisation programme] and longer 30/40-minute clinics for frailer patients with more symptoms (i.e., the palliative/complex pathway). Two weeks later, a face-to-face or phone consultation includes a second set of PROMs and CROMs [quality of life P2 and a C2]. Three months later, another set is collected [C3 and P3], typically occurring 6 months from the initial appointment.

Patients clinically well for a month are either discharged or referred to a Standard Clinic (up to 12 months). Upon discharge, all patients undergo a final set of PROMs and CROMs (sometimes occurring before the 6-month mark) and a Patient Reported Experience Measure (PREM).

- Patients with stable conditions self-manage symptoms, with re-referral to HF clinic if deterioration occurs.
- Referral to Community Care/Palliative Care, with communication maintained with the HF team as needed.
- Referral for Device Therapy involving implantable electronic devices.

**Parkinson’s Disease**

Parkinson's disease, a progressive neurological disorder primarily impacting movement, often manifests with common symptoms such as tremors, stiffness, or slowed movement. The Parkinson’s disease clinic, led by nurses, pioneered the testing of an implementation framework in 2016, becoming a trailblazer for potential expansion into other clinical areas. The principal objective in implementing Patient-Reported Outcome Measures (PROMs) was to leverage outcomes for delivering an efficient and effective service tailored to patient symptoms.

*Intervention:*

The care pathway, initially designed during the COVID-19 era and still in practice during this study, has adapted over time. Patients now undergo PROMs during their initial contact with the service and subsequently every six months. Face-to-face clinical appointments serve as occasions for PROMs completion, facilitated by Healthcare Assistants who assist patients either in-person or over the phone. Additionally, some patients opt for online PROMs completion at home, either independently or with support from family members.

**Process Maps for the PROMs intervention within the Heart Failure, Epilepsy, and Parkinson’s Services.**

**Figure A1.** Process Map for PROMs intervention within the Epilepsy Service


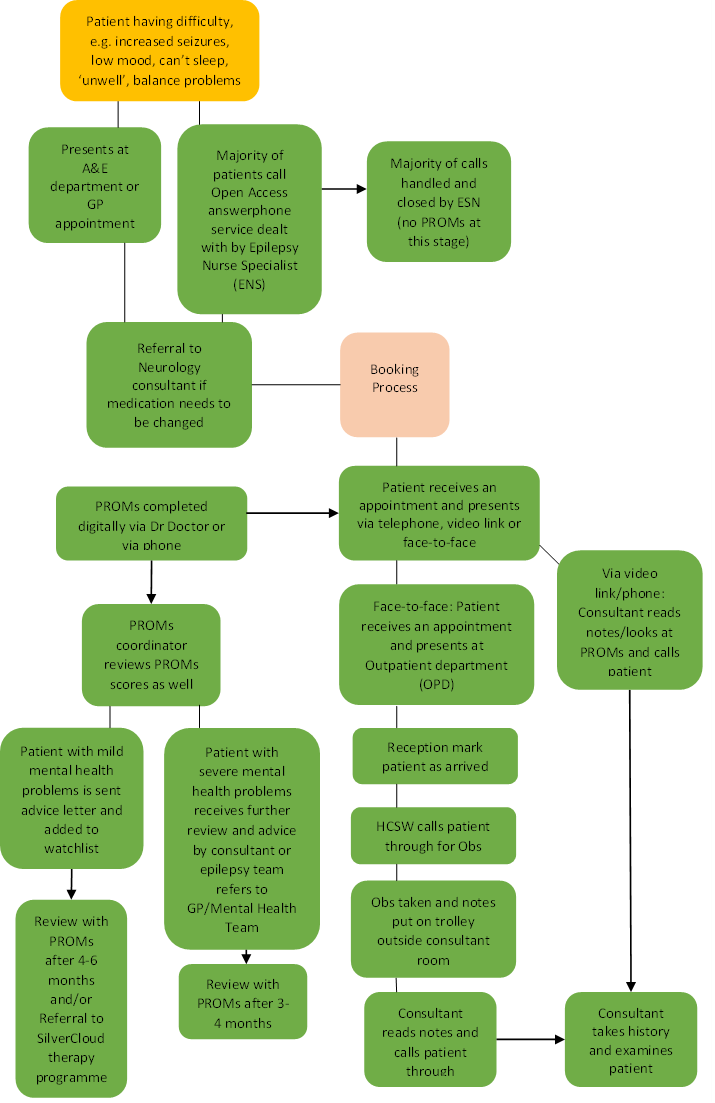


**Figure A2.** Process Map for PROMs intervention within the Heart Failure Service.

*Grey/White box = Process Step (patient present). White circle/diamond = Clinical decision. Blue box = PROM completed.*


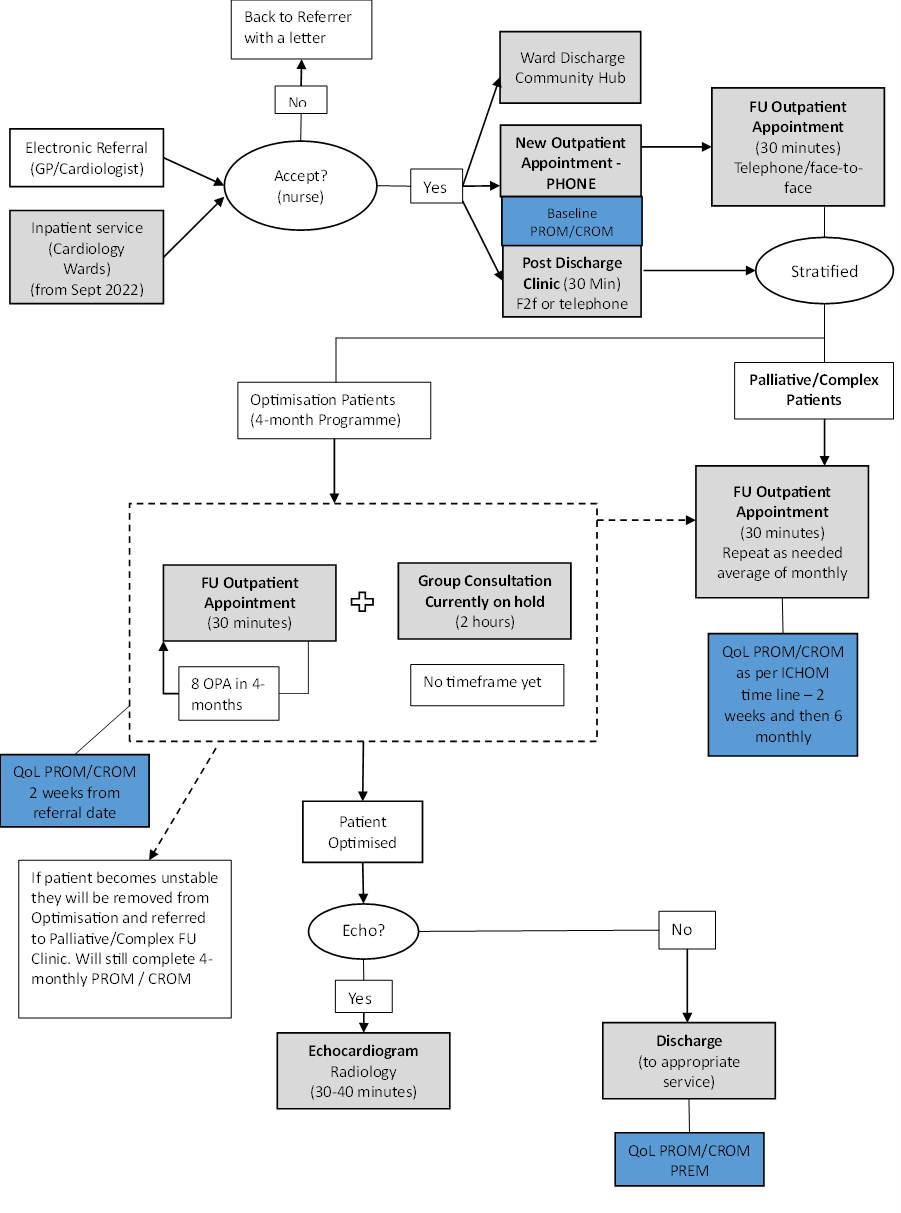


**Figure A3.** Process Map for PROMs intervention within the Parkinson’s Service.


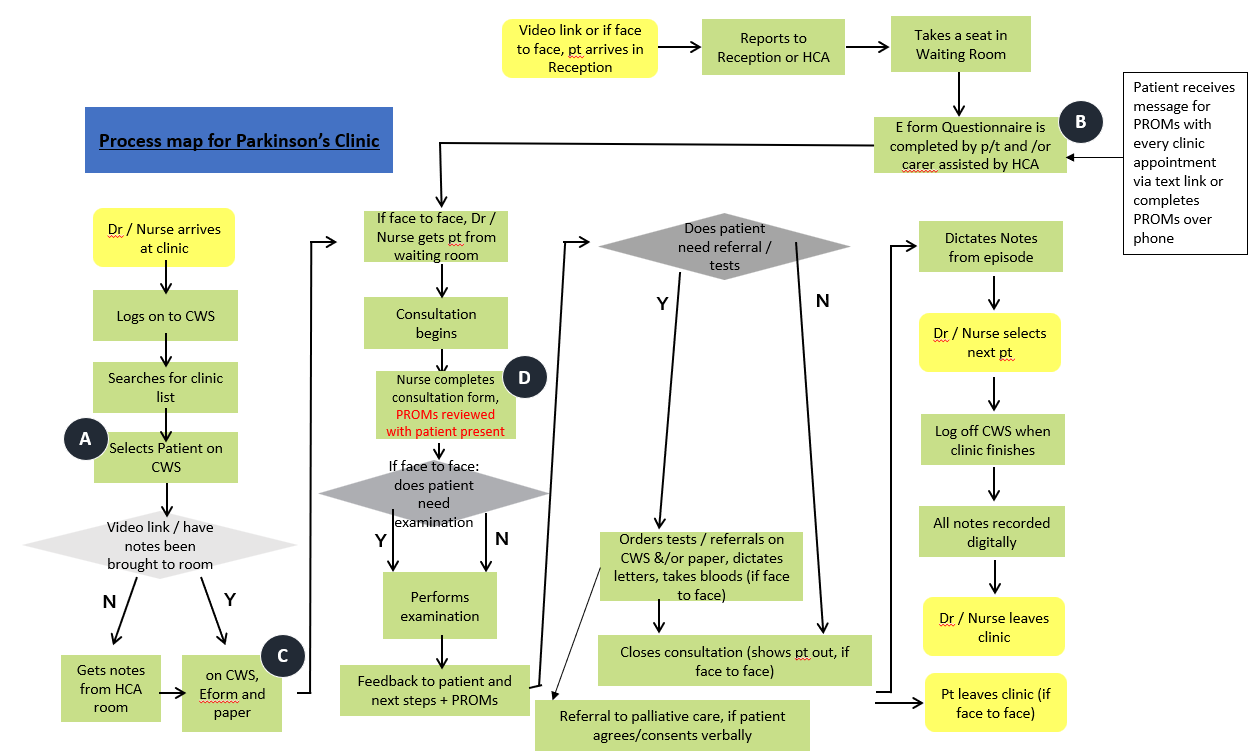


**Appendix 2:** Theory of Change Models

**Figure A4.** Theory of Change for PROMs intervention with the Epilepsy Service


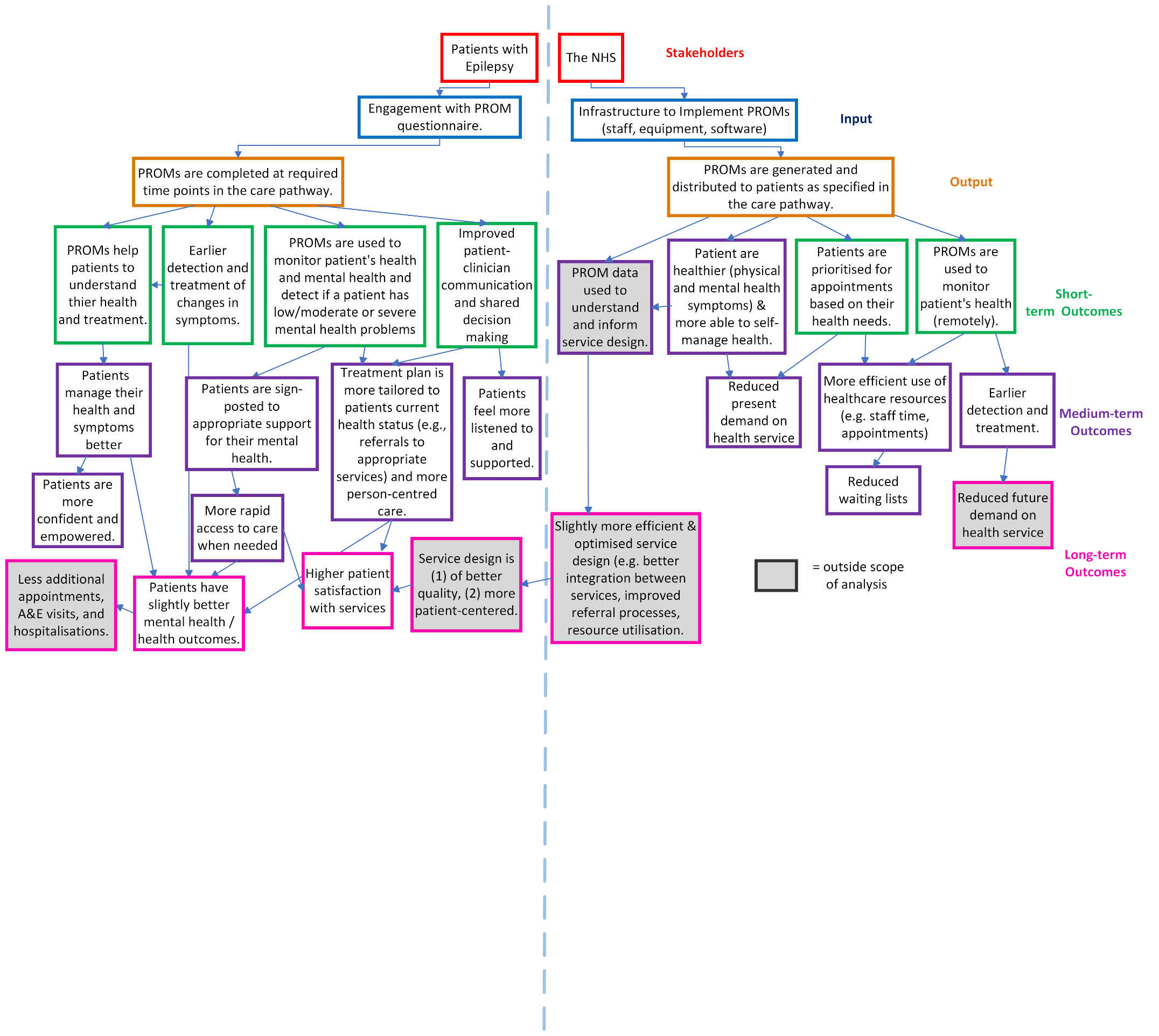


**Figure A5.** Theory of Change for PROMs intervention with the Heart Failure Service


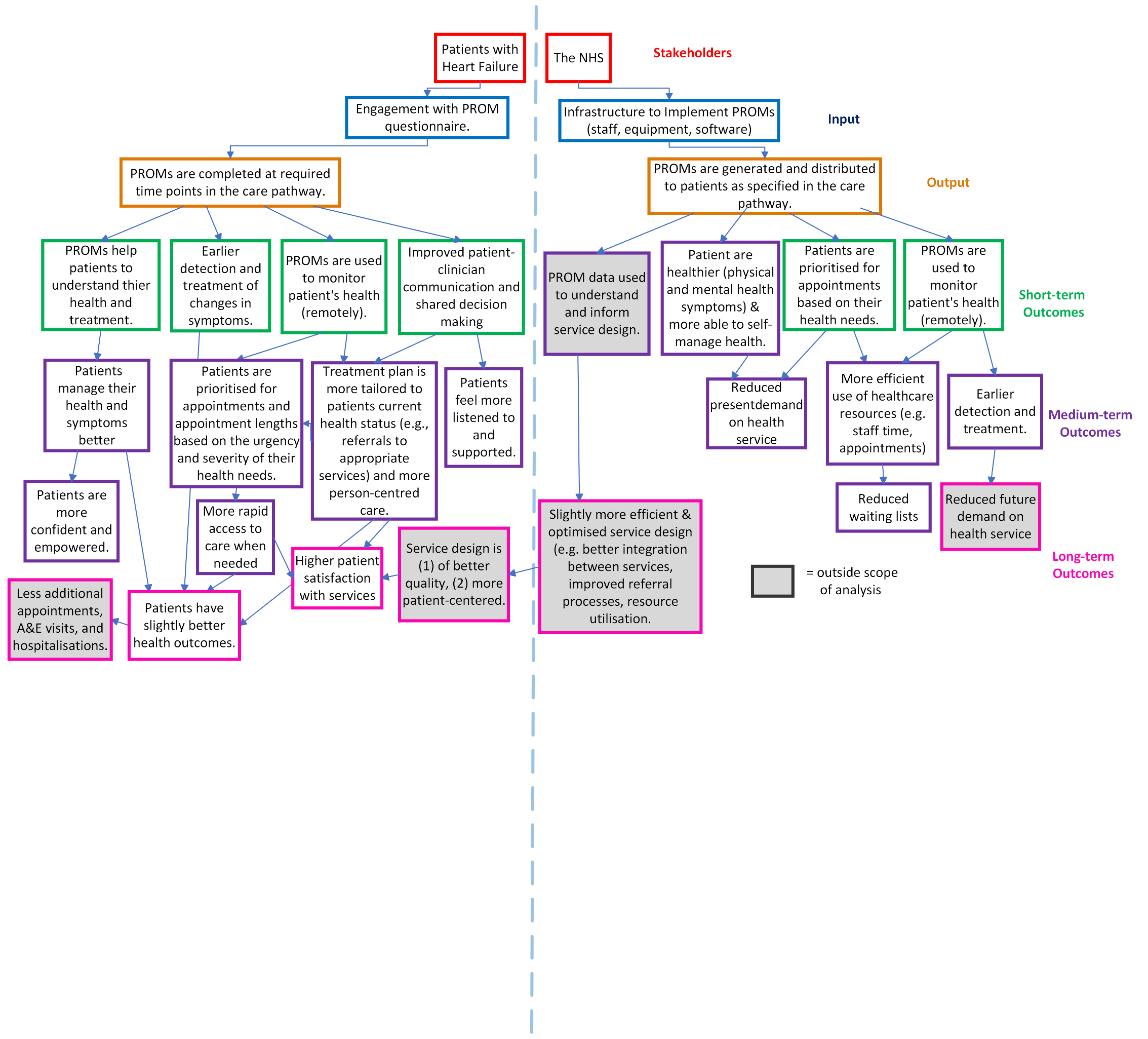


**Figure A6.** Theory of Change for PROMs intervention with the Parkinson’s Disease Service


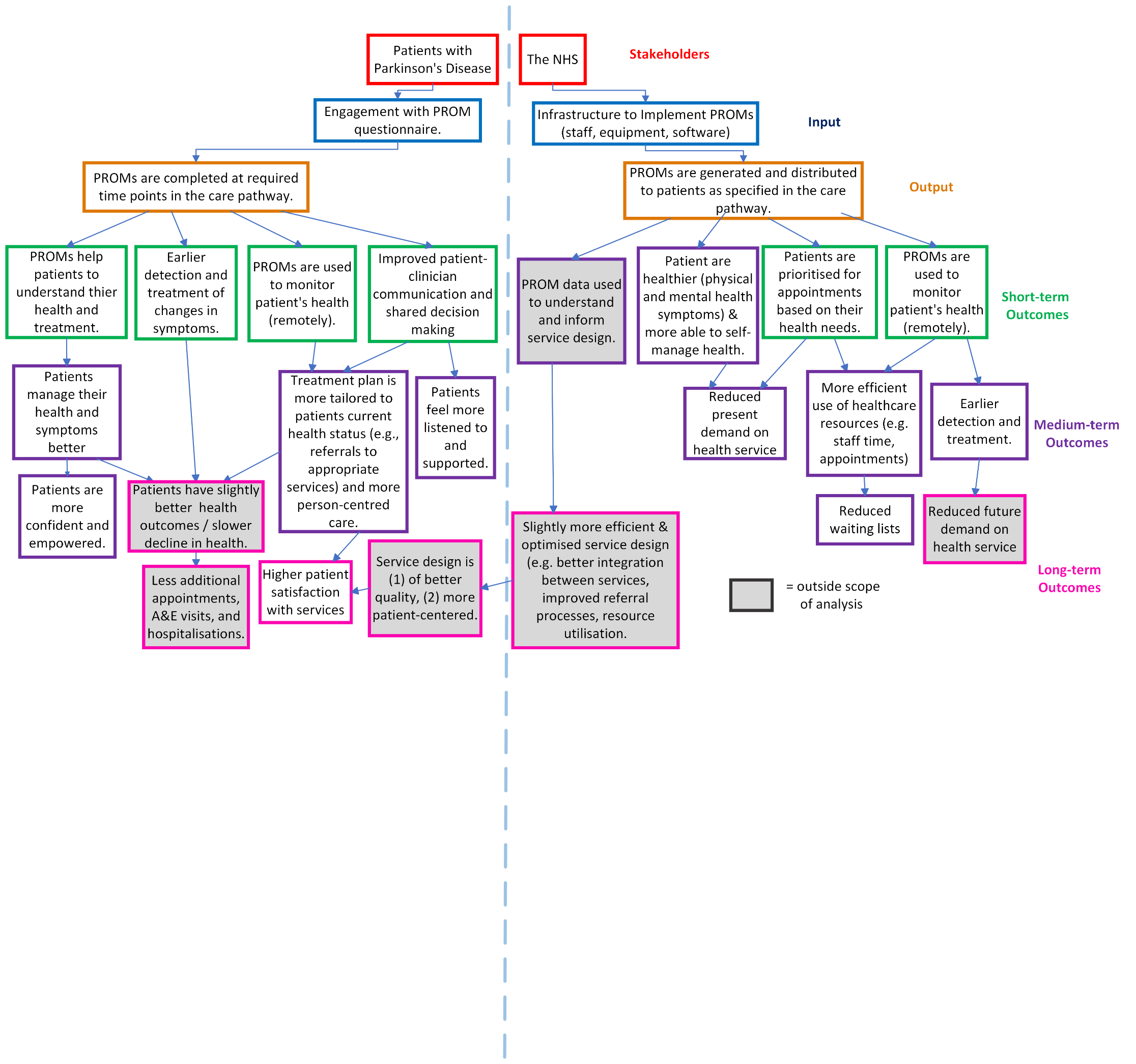


**Appendix 3:** Obtaining data for the SROI analyses.

**Table A1.** Data we sought to obtain for the SROI analysis, availability of data, and outcome.

| **Outcome** | **Data requested for this metric** | **Data Obtained** | **Outcome** |
| --- | --- | --- | --- |
| **Heart Failure** | | |  |
| Data to evidence outcome  Improved management of patients heart failure leads to slightly better health and health outcomes. | Inquired if there was any data on heart failure patient health outcome per annum. | No | We used questionnaire data to evidence this outcome. |
| Data to evidence outcome  Triage of patients improves service efficiency and leads to more rapid access to care when needed. | Waiting time for appointment per annum (either first appointment, or all appointments) per annum. | Yes | Data used to evidence outcome |
| Data to evidence outcome  Reduced present demand on health service. | Data on patient caseload per 1 fulltime equivalent staff member per annum.  or  Data on the number of outpatient appointments per patient in the heart failure service per annum. | Yes | Data used to evidence outcome. |
| Displacement  Reduced present demand on health service. | Data on the number of patients referred elsewhere within the NHS after triage at the heart failure service. | No | We utilised information from the realist analysis and stakeholder involvement to estimate this value. |
| Data to evidence outcome  Reduced future demand on health service. | Data on the number of cardiac related hospitalisations per heart failure patient per annum.  Data on the number of cardiac related A&E visits per heart failure patient per annum. | No | We had to remove this outcome from the analysis as the data we obtained was not able to adequately measure this metric. This was due to the lack of a comparator group of non-PROM completers. |
| **Epilepsy** | | |  |
| Data to evidence outcome  Improved management of patients epilepsy leads to slightly better epilepsy-related health. | Inquired if there was any data on epilepsy patient health outcome per annum. | No | We used questionnaire data to evidence this outcome. |
| Data to evidence outcome  Due to improved identification and signposting to mental health support, patients have improved mental health. | Data on the number of epilepsy patients from the PROMs service who have used the online mental health platform ‘SilverCloud’ per annum.  Data on the number of patients referred to the GP or to the Community Mental Health team per annum.  Data on the mental health outcomes for patients who complete the ‘SilverCloud’ course.  The number of mental health problems in epilepsy detected due to PROMs per annum. | No | We used questionnaire data to evidence this outcome. |
| Data to evidence outcome  Triage of patients improves service efficiency and leads to more rapid access to care when needed. | Waiting time for appointment per annum (either first appointment, or all appointments) per annum. | No | We had to remove this outcome from the analysis as we were not able to obtain data to adequately measure this metric. |
| Data to evidence outcome  Reduced present demand on health service. | Data on patient caseload per 1 fulltime equivalent staff member per annum.  or  Data on the number of outpatient appointments per patient in the epilepsy service per annum. | No | We had to remove this outcome from the analysis as we were not able to obtain data to adequately measure this metric. |
| Data to evidence outcome  Reduced future demand on health service. | Data on the number of mental health / seizure related hospitalisations per epilepsy patient per annum.  Data on the number of epilepsy related A&E visits per epilepsy patient per annum. | No | We had to remove this outcome from the analysis as the data we obtained was not able to adequately measure this metric. This was due to the lack of a comparator group of non-PROM completers. |
| **Parkinson’s** | | |  |
| Data to evidence outcome  Triage of patients improves service efficiency and leads to more rapid access to care when needed. | Waiting time for appointment per annum (either first appointment, or all appointments) per annum. | No | We had to remove this outcome from the analysis as we were not able to obtain data to adequately measure this metric. |
| Data to evidence outcome  Reduced present demand on health service. | Data on patient caseload per 1 fulltime equivalent staff member per annum.  or  Data on the number of outpatient appointments per patient in the Parkinson’s service per annum. | No | We had to remove this outcome from the analysis as we were not able to obtain data to adequately measure this metric. |
| Data to evidence outcome  Reduced future demand on health service. | Data on the number of hospitalisations per Parkinson’s patient per annum.  Data on the number of Parkinson’s related A&E visits per Parkinson’s patient per annum. | No | We had to remove this outcome from the analysis as the data we obtained was not able to adequately measure this metric. This was due to the lack of a comparator group of non-PROM completers. |

**Appendix 4.** Participant Information Sheets, Consent Form, Patient Questionnaires & Staff Questionnaires

**Participant information sheet for patient questionnaires**

Getting the Best from your NHS

Participant Information Sheet

You are being invited to take part in an online research study about the impact of Patient Reported Outcome Measures (PROMs) in your NHS care.

You may already have been interviewed for this study. If you have already been interviewed, we would still like you to fill out this questionnaire.

Before you decide whether you want to take part in the study, it is important for you to understand why this research is being done and what it would involve for you.

Please take your time to read through the following information and contact one of the researchers if you have any questions.

**Background**

Aneurin Bevan University Health Board (ABUHB) is the first health board in Wales to implement “Value Based Healthcare” – this is an approach to your care that tries to put what is important to you at the heart of all decisions about you and your care. One of the ways of doing this is through Patient Reported Outcome Measures (PROMs)

**What are Patient Reported Outcome Measures (PROMS)?**

Patient Reported Outcome Measures (PROMS) are a group of questions created for you to tell the Health Board about your health, experiences, and quality of life. PROMs help you and your healthcare team to monitor your progress and symptoms.

You may have been asked to complete a PROM as part of your *[CONDITION]*’s care. The PROM would have been given to you via phone, a text message, a letter, or an email.

**What is the purpose of the research?**

A research team from Bangor University are conducting an online study to measure the benefits that Patient Reported Outcome Measure Questionnaires (PROMs) provide for patients. For example, do PROMs help patients feel that their symptoms are being monitored better.

**Why are we asking for your help?**

We have contacted you because we want to hear about your experiences of healthcare in the NHS. We are also contacting you as you may have experience with Patient Reported Outcome Measures (PROMs).

Sharing your views and experiences will help us learn more about whether Patient Reported Outcome Measures (PROMs) are helping to improve standards of NHS care.

You can participate in this questionnaire regardless of whether you have ever completed a PROM as part of your healthcare.

**Who are we?**

We are a team of experienced researchers at Bangor University in partnership with the Aneurin Bevan University Health Board (ABUHB).

**Who is organizing and funding the research?**

The Welsh Government are funding this study. Aneurin Bevan University Health Board are the sponsor.

**Do I have to take part?**

It is completely up to you to decide if you want to take part in this study. You can withdraw at any time from the study without giving a reason.

**What will taking part in this research study involve?**

If you consent to take part in this study, you will be asked to complete an online questionnaire. This should take around **X minutes**.

The online questionnaire will start with some simple questions about yourself, such as your age and gender. After this, there will be some questions about your experiences of NHS healthcare and your experiences with PROMs. Some of these questions will ask you about your physical and mental health. All questions will be multiple choice. However, there is the option to provide further explanation for some questions if you wish to do so.

At the end of the questionnaire, there will be the option to follow a link to claim your £5 voucher. To obtain the voucher you will need to provide your email. To ensure your anonymity, your email will be stored separately from the information you provide in the questionnaire.

If you want to stop taking part during the study, you can exit at any point by closing the online browser window.

**What are the possible benefits of taking part?**

If you participate in this study, you will receive a £5 Amazon Voucher.

Participation in this study may not benefit you directly. However, the information we gain will help us to understand more about the benefits of Patient Reported Outcome Measures (PROMs). This will help NHS Wales to improve healthcare services for patients.

**What are the possible disadvantages and risks of taking part?**

We will ask about your health and experiences of your healthcare. The questions will not probe personal and difficult experiences, and all the answers are fixed format. Although it is unlikely that this will be distressing, be reassured that you can stop at any time.

If you have any worries about any of the points mentioned here, and you want to discuss this further, please contact one of the researchers for this study (contact details are at the end of this document).

**What if I have concerns about the study?**

It is important to know that you can voice your concerns or complaints about any aspect of this study. If something goes wrong during your participation, or you have a complaint, please contact the Chief Investigator, Dr Gareth Roberts via hayley.lewis@wales.nhs.uk

If the research team are unable to resolve your query and/or you would like to speak to somebody outside of the research team, you can contact the Aneurin Bevan Research Manager on [ABB.RandD@wales.nhs.](mailto:ABB.RandD@wales.nhs)uk

**Data protection information:**

**What will happen to the information I give?**

The data you provide in the questionnaire will be anonymized and analyzed by members of the research team at Bangor University. We will not share any of your identifiable details unless you tell us something that makes us seriously concerned about you or another person’s safety such as a child or vulnerable adult.

**What are your choices about how your information is used?**

As a publicly funded organization, we have to ensure that it is in the public interest when we use personally- identifiable information from people who have agreed to take part in research. This means that when you agree to take part in a research study, we will use your data in the ways needed to conduct and analyze the research study.

Your rights to access, change or move your information are limited, as we need to manage your information in specific ways in order for the research to be reliable and accurate. If you withdraw from the study, we will keep the information about you that we have already obtained. To safeguard your rights, we will use the minimum personally identifiable information possible.

 You can find out more about how we use your information:

- <https://www.hra.nhs.uk/information-about-patients/>
- By asking the research team
- By contacting the Data Protection Officer Jonathan Meredith at [DPO.ABB@wales.nhs.uk.](mailto:DPO.ABB@wales.nhs.uk)

**What will happen to the results of the research project?**

The study will be completed in December 2023. The findings from this study will be written up as an academic paper for submission to an academic journal. The findings will also be written up as a report for the Aneurin Bevan University Health Board.

A summary of the findings of the study will also be produced for patients and the public. You will be asked whether you want to be sent a copy of the research findings.

**Who can I speak to if I have more questions or worries?**

You will be able to download a copy of this Participant Information Sheet for your records.

If you have any further questions or would like assistance at any point during the study, please contact Ellena Crane ([e.crane@bangor.ac.uk](mailto:e.crane@bangor.ac.uk)), Dr Carys Jones ([c.jones@bangor.ac.uk](mailto:c.jones@bangor.ac.uk)) or Dr Gareth Roberts (hayley.lewis@ wales.nhs.uk)

Thank you for considering participating in this study. We are appreciative of your time and help with this project.

**Consent Form for Patient and Staff Questionnaire**

Thank you for reading the Participant Information Sheet and for considering taking part in this research study.

Please tick the box to indicate if you consent to each of the statements below:

- I confirm that I have read the Participant Information Sheet for the above study. I have had the opportunity to consider the information, ask questions and have had these answered satisfactorily.
- I understand that my participation is voluntary and that I am free to withdraw at any time
   without giving any reason.
- I understand that you will write a report about the study findings.
- I understand that the information I provide in this questionnaire will be held by the research team and that this information will be kept strictly confidential.
- I understand that the information collected about me may be used to support other research in the future and may be shared anonymously with other researchers.
- I agree to allow the information that I give to be used for educational purposes such as future MSc or PhD student research.
- I agree to take part in the above study.

*Participants will not be able to proceed with the study if they do not tick each box to indicate that they consent to the statement.*

Questionnaire Structure for patient and staff questionnaires

1. Demographic information
2. A ranking exercise ordering the outcomes by what the participant most prioritised in their care / their patients care (data from this exercise was excluded from the analysis due to poor data quality.
3. Likert response questions to measure how much each outcome had been achieved in the past two years.
4. Patient questionnaires only: A description of PROMs and a question asking if the patient remembers completing a PROM. Patients who answered that they don’t remember completing a PROM were re-directed to the end of the questionnaire. Patients who answer ‘yes’ or ‘not sure’ proceeded with the following questions.
5. Patient questionnaire only: Questions about PROMs (e.g., number of PROMs completed)
6. Likert response questions to measure how much PROMs have contributed to achieving the outcomes of interest in the past two years.

Questionnaire for Epilepsy Patients

**Questions about you**

1. What is your age?
   1. 18-35 years
   2. 36-50 years
   3. 51-70 years
   4. 71+ years
   5. Prefer not to say.
2. What is your gender?
   1. Male
   2. Female
   3. Other (Include free text box to describe_________)
   4. Prefer not to say.
3. How would you describe your ethnic group?
   1. Asian or Asian British
   2. Black, Black British, Caribbean, or African
   3. Mixed or multiple ethnic group
   4. White
   5. Prefer not to say.
   6. Other ethnic group (free text box _______)
4. What is your main language?
   1. English
   2. Welsh
   3. Prefer not to say.
   4. Other (include free text box to describe_____________)
5. Roughly, how many years has it been since you were diagnosed with epilepsy?
   1. Less than a year
   2. 1-2 years
   3. 3-5 years
   4. 6-10 years
   5. 10+ years
   6. Not sure

---------------------------- Page Break ----------------------------

For this section, we are interested in learning about your experiences of the NHS as a person who has Epilepsy.

Below is a list of statements.

Please rank the statements in order of what you most prioritise in your Epilepsy care, with 1 being the highest priority.

*You can drag and drop to rearrange the statements below.*

[For this question item, we used the Ranking Question function from Survey Monkey]

| My epilepsy being better controlled.  *Well-controlled epilepsy would mean that you have very few or no seizures.* |
| --- |
| My epilepsy treatment plan being more quickly adapted to changes in my health.  *For example, your clinician quickly changes your medication dosage if you start having more seizures.* |
| Mental health concerns being more quickly identified by your epilepsy healthcare team.  *Mental health concerns would include feelings of anxiety or low mood that have affected your wellbeing for at least two weeks.* |
| Having quicker access to mental health support.  *Support would include things like mental health therapy, or self-help guides.* |
| Having a better understanding and awareness of my epilepsy.  *For example, having a good understanding of your seizure triggers, medication side effects, or your mental health.* |
| Being able to better self-monitor my epilepsy symptoms  *For example, being able to keep track of how often you have a seizure, medication side-effects, mental health, or general health.* |
| Being listened to by my epilepsy healthcare team.  *For example, feeling that my clinician listens to and takes seriously my worries, priorities, or goals in my epilepsy care.* |
| Being more involved in making decisions about my epilepsy care.  For example, being involved in decisions about your epilepsy medication, or support needed to manage my day-to-day living. |

If you have further comments, or wish to expand upon your answers, please explain here.

Free text box _______

---------------------------- Page Break ----------------------------

The next set of questions is to find out **how much you have experienced** the following outcomes in your epilepsy care.

Please tick the box that best represents **your experience** of your epilepsy care in the past two years (or since your diagnosis if less than 2 years).

1. Please tick the box that best represents your experiences in the past two years as a person with epilepsy.

‘My epilepsy has been more well controlled.’

*Well-controlled epilepsy would mean that you have very few or no seizures.*

- *None of the time*
- *Rarely*
- *Often*
- *All of the time*
- *Not applicable*

*[Likert response options are repeated for all questions on this page]*

1. Please tick the box that best represents your experiences in the past two years as a person with epilepsy.

‘My epilepsy treatment plan has been more quickly adapted to changes in my health.’

*For example, your clinician quickly changes your medication dosage if you start having more seizures.*

1. Please tick the box that best represents your experiences in the past two years as a person with epilepsy.

‘Any mental health concerns have been more quickly identified by my epilepsy healthcare team.’

*Mental health concerns would include feelings of anxiety or low mood that have affected your wellbeing for at least two weeks.*

If this question is not relevant to you because you have never experienced any mental health worries, tick ‘not applicable’.

1. Please tick the box that best represents your experiences in the past two years as a person with epilepsy.

‘When mental health concerns have been identified by the epilepsy team, I have had quick access to mental health support.’

*Support would include things like mental health therapy, or self-help guides.*

If this question is not relevant to you, tick ‘not applicable’.

1. Please tick the box that best represents your experiences in the past two years as a person with epilepsy.

‘I have a better understanding and awareness of my epilepsy.’

*For example, having a good understanding of your seizure triggers, medication side effects, or your mental health.*

1. Please tick the box that best represents your experiences in the past two years as a person with epilepsy.

‘I have been better able to monitor my epilepsy symptoms.’

*For example, keeping track of how often you have a seizure, medication side-effects, or your mental health.*

1. Please tick the box that best represents your experiences in the past two years as a person with epilepsy.

‘In my epilepsy care, my clinicians have listened to me more.’

*For example, I felt that my clinician listened to and took seriously my worries, priorities, or goals in my epilepsy care.*

1. Please tick the box that best represents your experiences in the past two years as a person with epilepsy.

‘I have felt more involved in making decisions about my epilepsy care.’

*For example, being involved in decisions about your epilepsy medication, or support needed to manage your day-to-day living.*

If you have further comments, or wish to expand upon your answers, please explain here.

Free text box _______

---------------------------- Page Break ----------------------------

What are Patient Reported Outcomes Measures (PROMs)

Patient Reported Outcome Measures (PROMS) are a group of questions created for you and your healthcare team to assess and monitor your epilepsy symptoms and mental health. PROMs can also be used to help you and your healthcare team to better make decisions together about your healthcare.

PROMs are being used in epilepsy service to:

- Better monitor and act on changes in your epilepsy symptoms.
- Better monitor and act on changes in mental health symptoms.
- Better tailor your healthcare to your individual health and needs.
- Help patients be more informed and involved in their healthcare.

You may have been asked to complete a PROM questionnaire as part of your epilepsy care.

Here is an example of what the PROM questionnaire looks like:


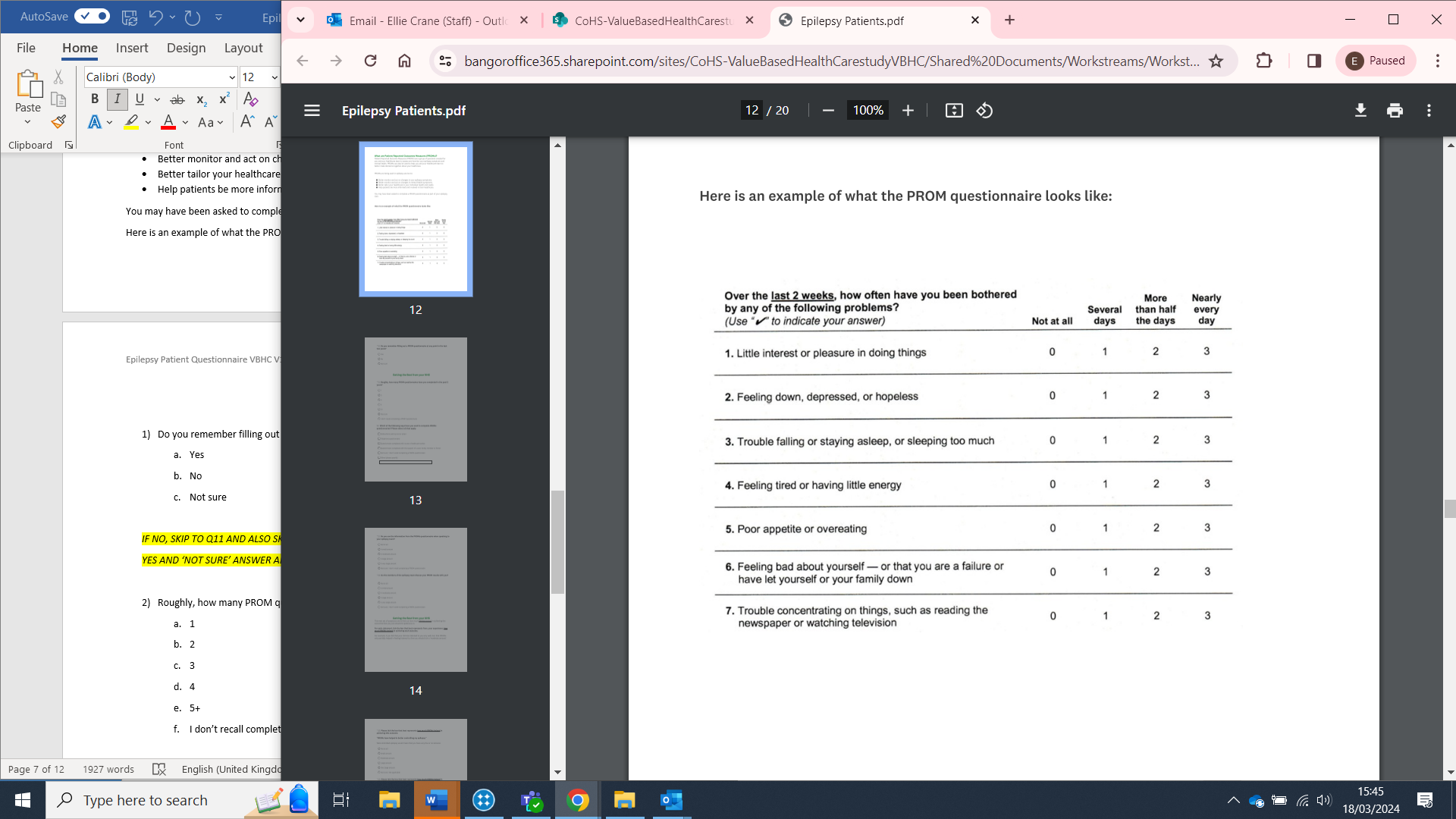


1. Do you remember filling out a PROM questionnaire at any point in the last two years?
   1. Yes
   2. No
   3. Not sure

*If participant answers ‘No’, they skip the remaining questions and are redirected to the end of the questionnaire.*

---------------------------- Page Break ----------------------------

Roughly, how many PROM questionnaires have you completed in the past 2 years?

- - 1
  - 2
  - 3
  - 4
  - 5+

I don’t recall completing a PROM questionnaire

Which of the following ways have you used to complete PROMs questionnaires? Please select all that apply.

Online form sent by text or email

Postal paper questionnaire

Telephone questionnaire

Paper questionnaire completed in clinic by yourself

Questionnaire completed with nurse or healthcare worker

Questionnaire completed with the support of a carer, family member or friend.

Not sure / I don’t recall completing a PROM questionnaire

Other (free text box _______)

Do you use the information from the PROMS questionnaire when speaking to your epilepsy team?

- - Not at all
  - A small amount
  - A moderate amount
  - A large amount

A very large amount

Not sure / I don’t recall completing a PROM questionnaire

Do the members of your epilepsy team discuss your PROM results with you?

- - Not at all
  - A small amount
  - A moderate amount
  - A large amount

A very large amount

Not sure / I don’t recall completing a PROM questionnaire

---------------------------- Page Break ----------------------------

This last set of questions are to find out how much PROMs helped in achieving the outcomes that you just answered questions on.

**For each statement, tick the box that best represents from your experience, how much PROMs helped in achieving each outcome.**

For example, if you felt that your clinician listened to you very well, but, that PROMs only partially helped in feeling listened to, then you should tick a ‘moderate amount’.

1. Please tick the box that best represents how much PROMs helped in achieving this outcome.

‘PROMs have helped in better controlling my epilepsy.’

*Well-controlled epilepsy would mean that you have very few or no seizures.*

- *Not at all*
- *Small amount*
- *Moderate amount*
- *Large amount*
- *Very large amount*
- *Not sure*
- *Not applicable*

*[Likert response options are repeated for all questions on this page]*

1. Please tick the box that best represents how much PROMs helped in achieving this outcome.

‘PROMs have helped with my epilepsy treatment plan being more quickly adapted to changes in my health. ‘

*For example, your clinician quickly changes your medication dosage if you start having more seizures.*

1. Please tick the box that best represents how much PROMs helped in achieving this outcome.

‘PROMs have helped mental health concerns be more quickly identified by my epilepsy healthcare team.

*Mental health concerns would include feelings of anxiety or low mood that have affected your wellbeing for at least two weeks.*

If this question is not relevant to you because you have never experienced any mental health worries, tick ‘not applicable’.

1. Please tick the box that best represents how much PROMs helped in achieving this outcome.

‘PROMs have helped with getting quicker access to mental health support.’

*Support would include things like mental health therapy, or self-help guides.*

If this question is not relevant to you, tick ‘not applicable’.

1. Please tick the box that best represents how much PROMs helped in achieving this outcome.

‘PROMs have help me to have a better understanding and awareness of my epilepsy.’

*For example, having a good understanding of your seizure triggers, medication side effects, or your mental health.*

1. Please tick the box that best represents how much PROMs helped in achieving this outcome.

‘PROMs have helped me to monitor my epilepsy symptoms.’

*For example, keeping track of how often you have a seizure, medication side-effects, or your mental health.*

1. Please tick the box that best represents how much PROMs helped in achieving this outcome.

‘In my epilepsy care, PROMS have helped in feeling more listened to by my clinician.’

*For example, I felt that my clinician listened to and took seriously my worries, priorities, or goals in my epilepsy care.*

1. Please tick the box that best represents how much PROMs helped in achieving this outcome.

‘PROMs have helped in being involved in making decisions about my epilepsy care.’

*For example, being involved in decisions about your epilepsy medication, or support needed to manage your day-to-day living.*

If you have further comments, or wish to expand upon your answers, please explain here.

Free text box _______

---------------------------- Page Break ----------------------------

Overall, to what extent do you think PROMs improve your epilepsy care?

- Not at all
- A small amount
- A moderate amount
- A large amount
- A very large amount

Not sure / I don’t recall completing a PROM questionnaire

To what extent do you think that your mental health and/or mental health care improved because of PROMs?

- Not at all
- A small amount
- A moderate amount
- A large amount
- A very large amount

Not sure / I don’t recall completing a PROM questionnaire

Would it make any difference to you if PROMS were removed from your epilepsy care?

- Not at all
- A small amount
- A moderate amount
- A large amount
- A very large amount

Not sure / I don’t recall completing a PROM questionnaire

Can you think of an example when PROMs have had an effect (either positive or negative) on your health or healthcare? *[Optional Question]*

- Free text box ________

Questionnaire for Heart Failure Patients

**Questions about you**

What is your age?

- 18-35 years
- 36-50 years
- 51-70 years
- 71+ years
- Prefer not to say.

What is your gender?

- Male
- Female
- Other (Include free text box to describe_________)
- Prefer not to say.

How would you describe your ethnic group?

- Asian or Asian British
- Black, Black British, Caribbean, or African
- Mixed or multiple ethnic group
- White
- Prefer not to say.
- Other ethnic group (free text box _______)

What is your main language?

- English
- Welsh
- Prefer not to say.
- Other (include free text box to describe_____________)

Roughly, how many years has it been since you were diagnosed with heart failure?

1. Less than a year
2. 1-2 years
3. 3-5 years
4. 6-10 years
5. 10+ years
6. Not sure

---------------------------- Page Break ----------------------------

For this section, we are interested in learning about your experiences of the NHS as a person who has Heart Failure.

Below is a list of statements.

Please rank the statements in order of what you most prioritise in your Heart Failure care, with 1 being the highest priority.

*You can drag and drop to rearrange the statements below.*

[For this question item, we used the Ranking Question function from Survey Monkey]

| Being better triaged to the best healthcare options based on my individual health and needs.  *For example, if your symptoms are worsening, you are given a longer appointment to meet your additional needs. Or, based on your symptoms, you are referred to a physiotherapy program for people with heart failure.* |
| --- |
| Being better listened to by my heart failure healthcare team.  *For example, feeling that your clinician listened to and took seriously your worries, priorities, or goals in your heart failure care.* |
| Being more informed and supported in achieving any individual health goals I have with my heart failure.  *For example, being referred to an exercise program to support a goal of being more physically active or being referred to occupational health to support a goal of maintaining independence as much as possible.* |
| Being more involved in making decisions about my heart failure care.  *For example, this could mean being involved in decisions about your medication options or being involved in planning support to maintain independence and quality of life.* |
| Having a better understanding and awareness of heart failure and my individual symptoms.  *For example, being aware of your individual heart failure symptoms and how they affect you.* |
| Being better able to self-monitor my heart failure symptoms.  *For example, keeping track overtime of any changes in your symptoms, how heart failure is affecting your quality of life and ability to carry out everyday tasks, or tracking medication side-effects.* |
| Feeling more motivated to take action to look after my health.  *For example, this could mean that you are being pro-active in managing your heart failure healthcare, taking steps to exercise, or getting more socially active in your community etc.* |
| Receiving more timely access to treatment for my heart failure |
| Having changes or complications in my heart failure symptoms identified and acted upon more quickly by my healthcare team.  *For example, your clinician is good at quickly addressing identified needs in your heart failure care.* |

If you have further comments, or wish to expand upon your answers, please explain here.

Free text box _______

---------------------------- Page Break ----------------------------

The next set of questions is to find out **how much you have experienced** the following outcomes in your heart failure care.

1. Please tick the box that best represents your experience of your heart failure care in the past two years (or since your diagnosis if less than 2 years).

‘I have been better triaged to the best healthcare options based on my individual health and needs.’

*For example, if your symptoms are worsening, you are given a longer appointment to meet your additional healthcare needs. Or, based on your symptoms, you are referred to a physiotherapy program for people with heart failure.*

- *None of the time*
- *Rarely*
- *Often*
- *All of the time*
- *Not applicable*

*[Likert response options are repeated for all questions on this page]*

1. Please tick the box that best represents your experience of your heart failure care in the past two years (or since your diagnosis if less than 2 years).

‘In my heart failure care, my clinicians have listened to me more. ‘

*For example, feeling that your clinician listened to and took seriously your worries, priorities, or goals in your heart failure care.*

1. Please tick the box that best represents your experience of your heart failure care in the past two years (or since your diagnosis if less than 2 years).

‘I have felt more informed and supported in achieving any individual health goals I have with my heart failure.’

*For example, being referred to an exercise program to support a goal of being more physically active or being referred to occupational therapy to support a goal of maintaining independence as much as possible.*

1. Please tick the box that best represents your experience of your heart failure care in the past two years (or since your diagnosis if less than 2 years).

‘I have felt more involved in making decisions about my heart failure.’

*For example, this could mean being involved in decisions about your medication options or being involved in planning support to maintain independence and quality of life.*

1. Please tick the box that best represents your experience of your heart failure care in the past two years (or since your diagnosis if less than 2 years).

‘I have a better understanding and awareness of heart failure and my individual symptoms.’

*For example, being aware of your individual heart failure symptoms and how they affect you.*

1. Please tick the box that best represents your experience of your heart failure care in the past two years (or since your diagnosis if less than 2 years).

‘I have been better able to monitor my heart failure symptoms.’

*For example, keeping track overtime of any changes in your symptoms, how heart failure is affecting your quality of life and ability to carry out everyday tasks, or tracking medication side-effects.*

1. Please tick the box that best represents your experience of your heart failure care in the past two years (or since your diagnosis if less than 2 years).

‘I have felt more motivated to take action to look after my health.’

*For example, this could mean that you are being pro-active in managing your healthcare, taking steps to exercise, or getting more socially active in your community etc.*

1. Please tick the box that best represents your experience of your heart failure care in the past two years (or since your diagnosis if less than 2 years).

‘I have received more timely access to treatment and support for my heart failure.’

1. Please tick the box that best represents your experience of your heart failure care in the past two years (or since your diagnosis if less than 2 years).

‘Changes or complications in my heart failure symptoms have been identified and acted upon more quickly by my healthcare team.’

*For example, your clinician is good at quickly addressing identified needs in your heart failure care.*

If you have further comments, or wish to expand upon your answers, please explain here.

Free text box _______

---------------------------- Page Break ----------------------------

What are Patient Reported Outcomes Measures (PROMs)

Patient Reported Outcome Measures (PROMS) are a group of questions created for you and your healthcare team to assess and monitor your heart failure symptoms. PROMs can also be used to help you and your healthcare team to better make decisions together about your healthcare.

PROMs are being used in heart failure services to:

- Better monitor and act on changes in your heart failure symptoms.
- Better tailor your healthcare to your individual health and needs.
- Help patients be more informed and involved in their care.

You may have been asked to complete a PROM questionnaire as part of your heart failure care.

Here is an example of what the PROM questionnaire looks like:


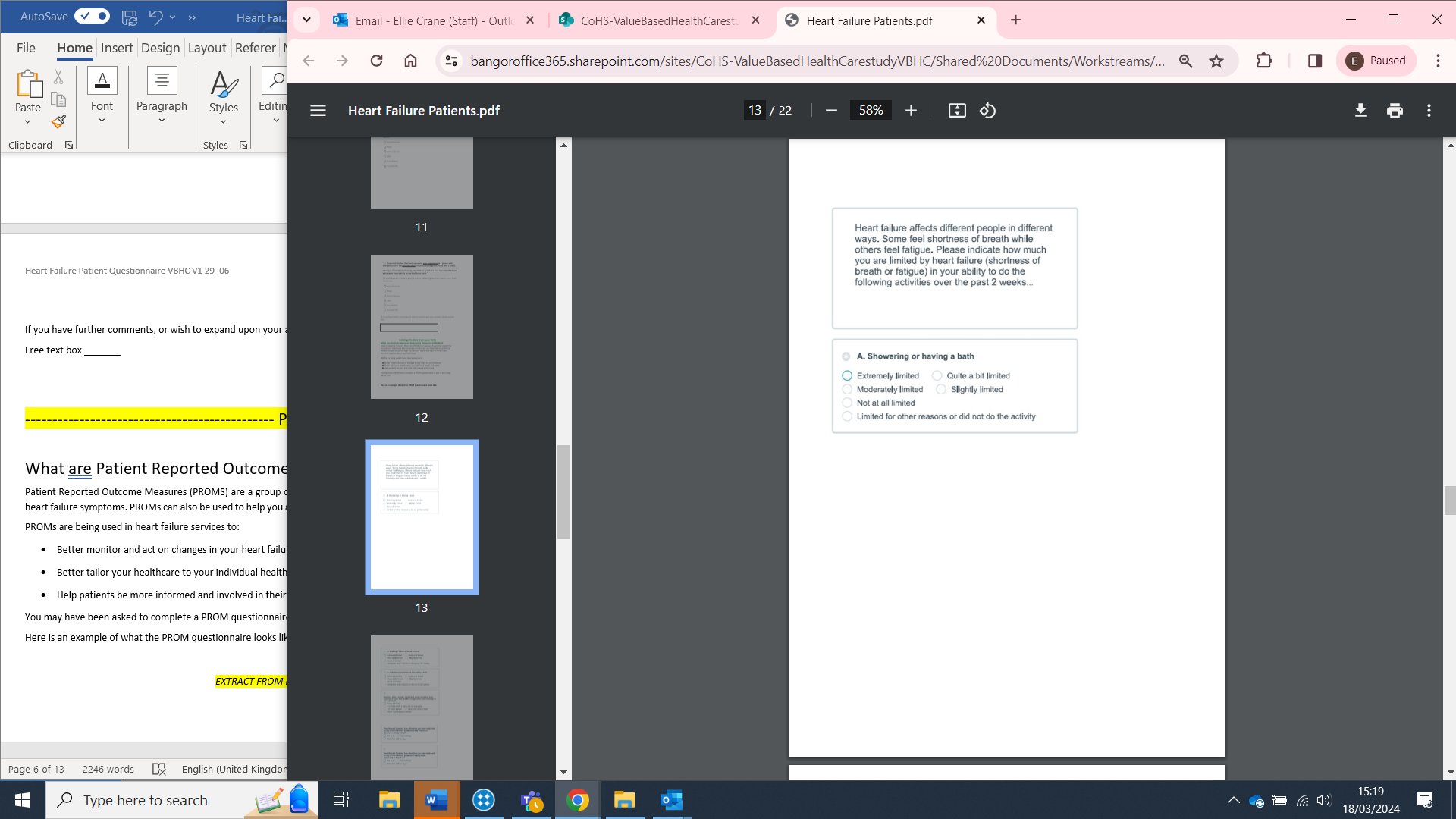


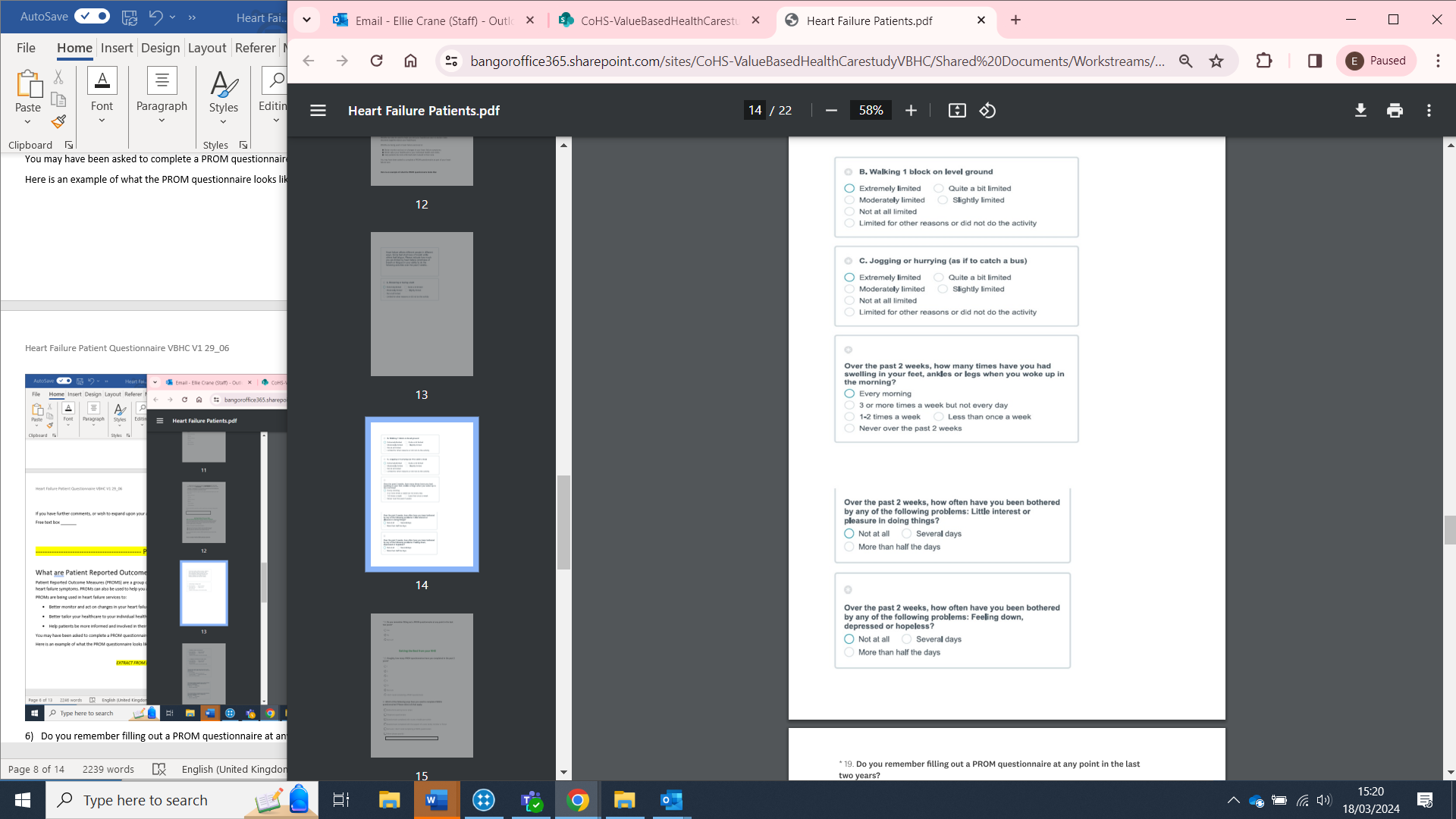


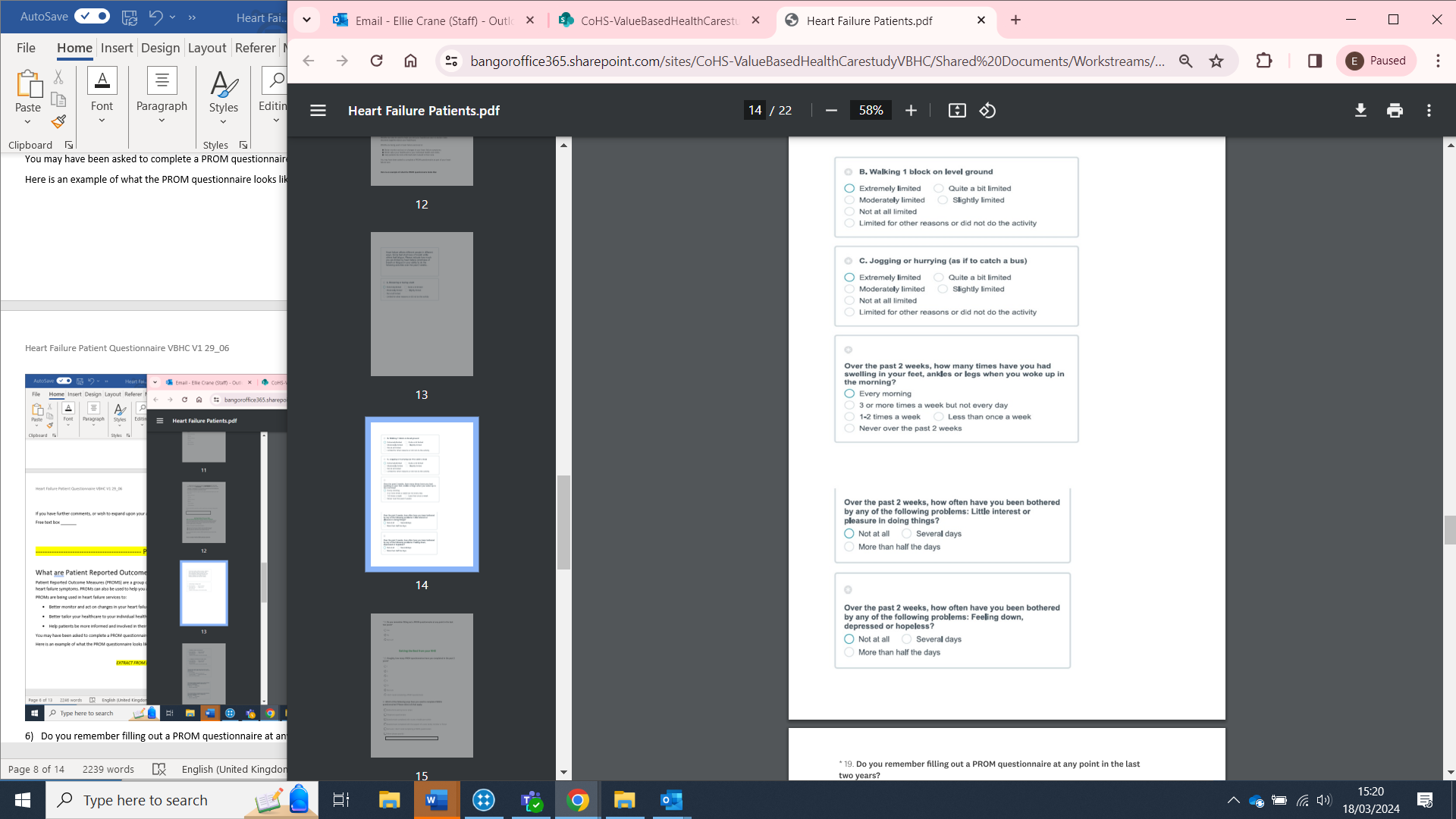


Do you remember filling out a PROM questionnaire at any point in the last two years?

- 1. Yes
  2. No
  3. Not sure

*If participant answers ‘No’, they skip the remaining questions and are redirected to the end of the questionnaire.*

---------------------------- Page Break ----------------------------

Roughly, how many PROM questionnaires have you completed in the past 2 years?

- - 1
  - 2
  - 3
  - 4
  - 5+

Not sure

I don’t recall completing a PROM questionnaire

Which of the following ways have you used to complete PROMs questionnaires? Please select all that apply.

Online form sent by text or email

Postal paper questionnaire

Telephone questionnaire

Paper questionnaire completed in clinic by yourself

Questionnaire completed with nurse or healthcare worker

Questionnaire completed with the support of a carer, family member or friend.

Not sure / I don’t recall completing a PROM questionnaire

Other (free text box _______)

Do you use the information from the PROMs questionnaire when speaking to your heart failure team?

- - Not at all
  - A small amount
  - A moderate amount
  - A large amount

A very large amount

Not sure / I don’t recall completing a PROM questionnaire

Do the members of the heart failure team discuss your PROM results with you?

- - Not at all
  - A small amount
  - A moderate amount
  - A large amount

A very large amount

Not sure / I don’t recall completing a PROM questionnaire

---------------------------- Page Break ----------------------------

This last set of questions are to find out how much PROMs helped in achieving the outcomes that you just answered questions on.

**For each statement, tick the box that best represents from your experience, how much PROMs helped in achieving each outcome.**

For example, if you felt that your clinician listened to you very well, but, that PROMs only partially helped in feeling listened to, then you should tick a ‘moderate amount’.

1. Please tick the box that best represents how much PROMs helped in achieving this outcome.

‘PROMs have helped me in being better triaged to the best healthcare options based on my individual health and needs.’

*For example, if your symptoms are worsening, you are given a longer appointment to meet your additional needs. Or, based on your symptoms, you are referred to a physiotherapy program for people with heart failure.*

- *Not at all*
- *Small amount*
- *Moderate amount*
- *Large amount*
- *Very large amount*
- *Not sure*
- *Not applicable*

*[Likert response options are repeated for all questions on this page]*

1. Please tick the box that best represents how much PROMs helped in achieving this outcome.

‘In my heart failure care, PROMS have helped me in feeling more listened to by my clinician.’

*For example, feeling that your clinician listened to and took seriously your worries, priorities, or goals in your heart failure care.*

1. Please tick the box that best represents how much PROMs helped in achieving this outcome.

‘PROMs have helped me in being more informed and supported in achieving any individual health goals I have with my heart failure.’

*For example, being referred to an exercise program to support a goal of being more physically active, or, being referred to occupational health to support a goal of maintaining more independence.*

1. Please tick the box that best represents how much PROMs helped in achieving this outcome.

‘PROMs have helped me in being involved in making decisions about my heart failure care.’

*For example, this could mean being involved in decisions about your medication options or being involved in planning support to maintain independence and quality of life.*

1. Please tick the box that best represents how much PROMs helped in achieving this outcome.

‘PROMs have helped me to have a good understanding and awareness of heart failure and my individual symptoms.’

*For example, being aware of your individual heart failure symptoms and how they affect you.*

1. Please tick the box that best represents how much PROMs helped in achieving this outcome.

‘PROMs have helped me to monitor my heart failure symptoms.’

*For example, keeping track overtime of any changes in your symptoms, how heart failure is affecting your quality of life and ability to carry out everyday tasks, or tracking medication side-effects.*

Please tick the box that best represents how much PROMs helped in achieving this outcome.

‘PROMs have helped me feel more motivated to take action to look after my health ‘

*For example, this could mean that you are being pro-active in managing your healthcare, taking steps to exercise, or getting more socially active in your community etc.*

1. Please tick the box that best represents how much PROMs helped in achieving this outcome.

‘PROMs have helped me to receive more timely access to treatment and support for my heart failure.’

1. Please tick the box that best represents how much PROMs helped in achieving this outcome.

‘PROMs have helped with changes or complications in my heart failure health being identified and acted upon more quickly.’

*For example, your clinician is good at quickly addressing identified needs in your Heart Failure care.*

If you have further comments, or wish to expand upon your answers, please explain here.

Free text box _______

---------------------------- Page Break ----------------------------

Overall, to what extent do you think PROMs improve your heart failure care?

- - Not at all
  - A small amount
  - A moderate amount
  - A large amount
  - A very large amount

Not sure / I don’t recall completing a PROM questionnaire

Would it make any difference to you if PROMS were removed from your heart failure care?

- - Not at all
  - A small amount
  - A moderate amount
  - A large amount
  - A very large amount

Not sure / I don’t recall completing a PROM questionnaire

Can you think of an example when PROMs have had an effect (either positive or negative) on your health or healthcare? *[Optional Question]*

- Free text box ________

Questionnaire for Parkinson’s Patients

**Questions about you**

1. What is your age?
   1. 18-35 years
   2. 36-50 years
   3. 51-70 years
   4. 71+ years
   5. Prefer not to say.
2. What is your gender?
   1. Male
   2. Female
   3. Other (Include free text box to describe_________)
   4. Prefer not to say.
3. How would you describe your ethnic group?
   1. Asian or Asian British
   2. Black, Black British, Caribbean, or African
   3. Mixed or multiple ethnic group
   4. White
   5. Prefer not to say.
   6. Other ethnic group (free text box _______)
4. What is your main language?
   1. English
   2. Welsh
   3. Prefer not to say.
   4. Other (include free text box to describe_____________)
5. Roughly, how many years has it been since you were diagnosed with Parkinson’s?
   1. Less than a year
   2. 1-2 years
   3. 3-5 years
   4. 6-10 years
   5. 10+ years
   6. Not sure
   7. Prefer not to say.

---------------------------- Page Break ----------------------------

For this section, we are interested in learning about your experiences of the NHS as a person who has Parkinson’s.

Below is a list of statements.

Please rank the statements in order of what you most prioritise in your Parkinson’s care, with 1 being the highest priority.

*You can drag and drop to rearrange the statements below.*

[For this question item, we used the Ranking Question function from Survey Monkey]

| Being better listened to by my Parkinson’s healthcare team. *E.g. feeling that your clinician listened to your worries, priorities, or goals in your Parkinson’s care.* |
| --- |
| Being more informed and supported in achieving any individual health goals I have with my Parkinson’s. *E.g., being provided with the information or help needed to support your goals of being physically active, or to maintain your independence with day-to-day living.* |
| Being more involved in making decisions about my Parkinson’s care. *E.g. being involved in decisions about your medication or referrals.* |
| Having a better understanding and awareness of Parkinson’s and my individual symptoms. *E.g. being aware of your individual motor and non-motor symptoms and how they affect you.* |
| Being better able to self-monitor my Parkinson’s symptoms. *E.g. keeping track overtime of any changes in your symptoms, medication effectiveness, or medication side-effects.* |
| Being more motivated to take action to look after my health. *E.g. being pro-active in managing your health, getting involved in physical activity, or being active in your community etc.* |
| My Parkinson’s treatment plan being more quickly adapted to changes in my health. *E.g. your clinician picks up on changes in your health and quickly changes your Parkinson’s medication to manage this.* |
| Being better triaged to the best healthcare options based on my individual health and needs. *E.g. if your symptoms are worsening, you are given a longer appointment to meet your additional needs.* |

If you have further comments, or wish to expand upon your answers, please explain here.

Free text box _______

---------------------------- Page Break ----------------------------

The next set of questions is to find out **how much you have experienced** in your Parkinson’s care the following outcomes.

**Please tick the box that best represents your experience of your Parkinson’s care in the past two years (or since your diagnosis if less than 2 years).**

1. Please tick the box that best represents **your experiences** as a person with Parkinson’s over the past two years.

“In my Parkinson’s care, my clinicians have listened to me more.”

*For example, feeling that your clinician listened to and took seriously your worries, priorities, or goals in your Parkinson’s care.*

- *None of the time*
- *Rarely*
- *Often*
- *All of the time*
- *Not applicable*

*[Likert response options are repeated for all questions on this page]*

1. Please tick the box that best represents **your experiences** as a person with Parkinson’s over the past two years.

“I have felt more informed and supported in achieving any individual health goals I have with my Parkinson’s.”

*For example, being provided resources to support your goals of being physically active, or support to maintain your independence.*

1. Please tick the box that best represents **your experiences** as a person with Parkinson’s over the past two years.

“I have felt more involved in making decisions about my Parkinson’s care.”

*For example, being involved in decisions about your medication, additional support such as speech and language therapy, or the support needed to manage your day-to-day living.*

1. Please tick the box that best represents **your experiences** as a person with Parkinson’s over the past two years.

*”* I have a better understanding and awareness of Parkinson’s and my individual symptoms.*”*

*For example, being aware of your individual motor and non-motor symptoms and how they affect you.*

1. Please tick the box that best represents **your experiences** as a person with Parkinson’s over the past two years.

*”* I have been better able to monitor my Parkinson’s symptoms.*”*

*For example, keeping track overtime of any changes in your symptoms, medication effectiveness, or medication side-effects.*

1. Please tick the box that best represents **your experiences** as a person with Parkinson’s over the past two years.

*”* I have felt more motivated to take action to look after my health.*”*

*For example, being pro-active in managing your health, getting involved in physical activity, or being more active in your community etc.*

1. Please tick the box that best represents **your experiences** as a person with Parkinson’s over the past two years.

*”* My Parkinson’s treatment plan has been more quickly adapted to changes in my health.*”*

*For example, your clinician picks up on changes in your health and quickly changes your Parkinson’s medication to manage this.*

1. Please tick the box that best represents **your experiences** as a person with Parkinson’s over the past two years.

I have been better triaged to the best healthcare options based on my individual health and needs.

*“For example, if your symptoms are worsening, you are given a longer appointment to meet your additional needs. Or, based on your symptoms, you are referred to a physiotherapy programme for people with Parkinson’s.”*

If you have further comments, or wish to expand upon your answers, please explain here.

Free text box _______

---------------------------- Page Break ----------------------------

What are Patient Reported Outcomes Measures (PROMs)

Patient Reported Outcome Measures (PROMS) are a group of questions created for you and your healthcare team to assess and monitor your Parkinson’s symptoms. PROMs can also be used to help you and your healthcare team to better make decisions together about your healthcare.

PROMs are being used in Parkinson’s services to:

- Better monitor and act on changes in your Parkinson’s symptoms.
- Better tailor your healthcare to your individual health and needs.
- Help patients be more informed and involved in their care.

You may have been asked to complete a PROM questionnaire as part of your Parkinson’s care.

Here is an example of what the PROM questionnaire looks like:


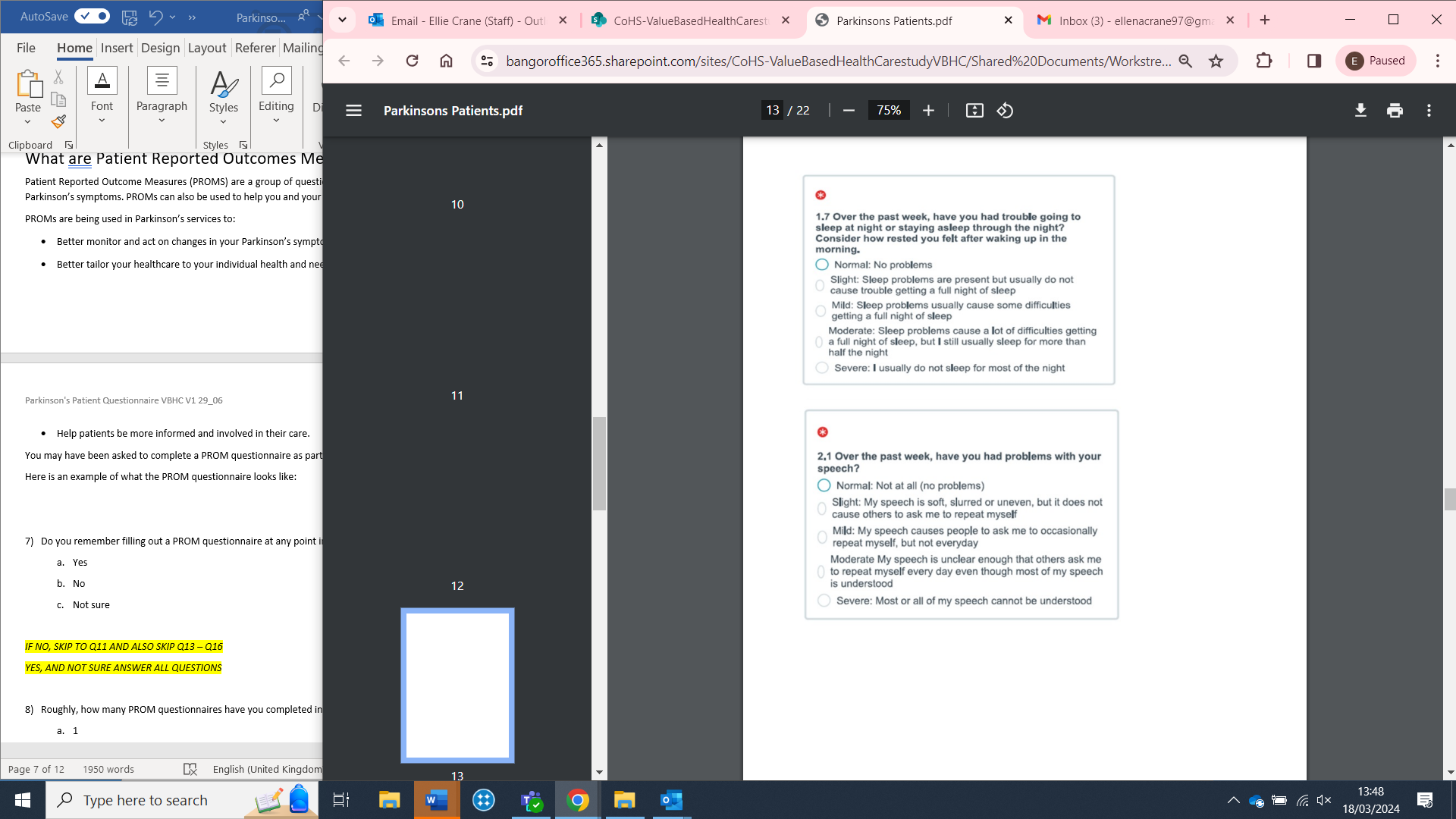


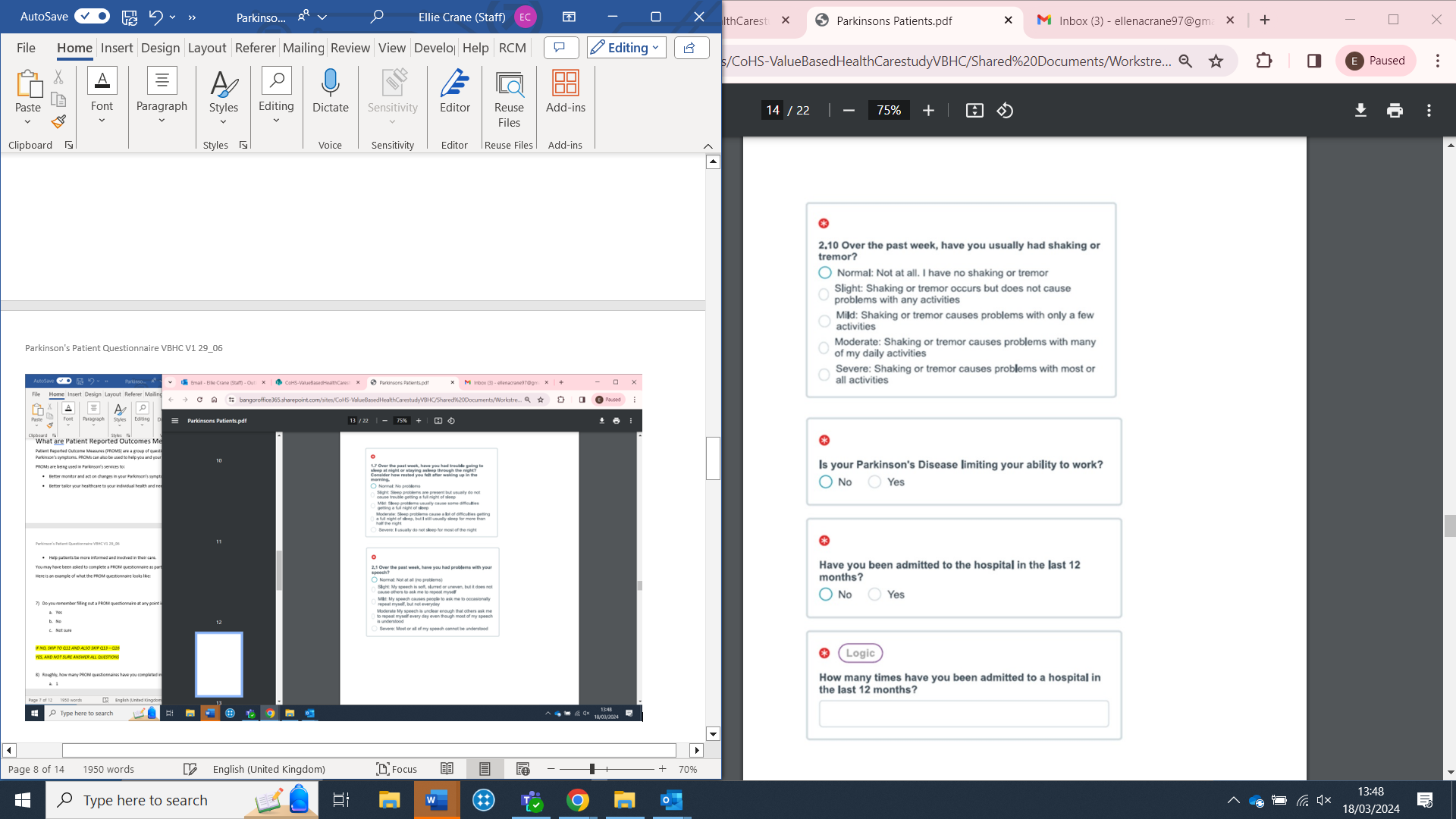


Do you remember filling out a PROM questionnaire at any point in the last two years?

- - Yes
  - No
  - Not sure

*If participant answers ‘No’, they skip the remaining questions and are redirected to the end of the questionnaire.*

---------------------------- Page Break ----------------------------

Roughly, how many PROM questionnaires have you completed in the past 2 years?

- - 1
  - 2
  - 3
  - 4
  - 5+

Not sure

I don’t recall completing a PROM questionnaire

Which of the following ways have you used to complete PROMs questionnaires? Please select all that apply.

Online form sent by text or email

Postal paper questionnaire

Telephone questionnaire

Paper questionnaire completed in clinic by yourself

Questionnaire completed with nurse or healthcare worker

Questionnaire completed with the support of a carer, family member or friend.

Not sure / I don’t recall completing a PROM questionnaire

Other (free text box _______)

Do you use the information from the PROMs questionnaire when speaking to your clinical Parkinson’s team?

- - Not at all
  - A small amount
  - A moderate amount
  - A large amount

A very large amount

Not sure / I don’t recall completing a PROM questionnaire

Do the members of your Parkinson’s healthcare team discuss your PROM results with you?

- - Not at all
  - A small amount
  - A moderate amount
  - A large amount

A very large amount

Not sure / I don’t recall completing a PROM questionnaire

---------------------------- Page Break ----------------------------

This last set of questions are to find out how much PROMs helped in achieving the outcomes that you just answered questions on.

**For each statement, tick the box that best represents from your experience, how much PROMs helped in achieving each outcome.**

For example, if you felt that your clinician listened to you very well, but, that PROMs only partially helped in feeling listened to, then you should tick a ‘moderate amount’.

1. Please tick the box that best represents how much PROMs helped in achieving this outcome.

‘In my Parkinson’s care, PROMs have helped me in feeling more listened to by my clinician.’

*For example, feeling that your clinician listened to and took seriously your worries, priorities, or goals in your Parkinson’s care.*

- *Not at all*
- *Small amount*
- *Moderate amount*
- *Large amount*
- *Very large amount*
- *Not sure*
- *Not applicable*

*[Likert response options are repeated for all questions on this page]*

1. Please tick the box that best represents how much PROMs helped in achieving this outcome.

‘PROMs have helped me in being more informed and supported in achieving any individual health goals I have with my Parkinson’s.’

*For example, being provided resources to support your goal of being physically active, or support to maintain your independence.*

1. Please tick the box that best represents how much PROMs helped in achieving this outcome.

‘PROMs have helped me in being more involved in making decisions about my Parkinson’s care.’

*For example, being involved in decisions about your medication, additional support such as speech and language therapy, or the support needed to manage your day-to-day living.*

1. Please tick the box that best represents how much PROMs helped in achieving this outcome.

‘PROMs have helped me to have a better understanding and awareness of Parkinson’s and my individual symptoms.’

*For example, being aware of your individual motor and non-motor symptoms and how they affect you.*

1. Please tick the box that best represents how much PROMs helped in achieving this outcome.

‘PROMs help me to better monitor my Parkinson’s symptoms.’

*For example, keeping track overtime of any changes in your symptoms, medication effectiveness, or medication side-effects.*

1. Please tick the box that best represents how much PROMs helped in achieving this outcome.

‘PROMs have helped me feel more motivated to take action to look after my health.’

*For example, being pro-active in managing your health, getting involved in physical activity, or being more active in your community etc*

1. Please tick the box that best represents how much PROMs helped in achieving this outcome.

‘PROMs have helped with my Parkinson’s treatment plan being more quickly adapted to changes in my health.’

*For example, your clinician picks up on changes in your health and quickly changes your Parkinson’s medication to manage this.*

1. Please tick the box that best represents how much PROMs helped in achieving this outcome.

‘PROMs have helped me in being better triaged to the best healthcare options based on my individual health and needs.’

*For example, if your symptoms are worsening, you are given a longer appointment to meet your additional needs. Or, based on your symptoms, you are referred to a physiotherapy programme for people with Parkinson’s.*

If you have further comments, or wish to expand upon your answers, please explain here.

Free text box _______

---------------------------- Page Break ----------------------------

Overall, to what extent do you think PROMs improve your Parkinson’s care?

- - Not at all
  - A small amount
  - A moderate amount
  - A large amount
  - A very large amount
  - Not sure / I don’t recall completing a PROM questionnaire

Would it make any difference to you if PROMS were removed from your Parkinson’s care?

- - Not at all
  - A small amount
  - A moderate amount
  - A large amount
  - A very large amount

Not sure / I don’t recall completing a PROM questionnaire

Can you think of an example when PROMs have had an effect (either positive or negative) on your health or healthcare? *[Optional Question]*

- - Free text box ________

Participant information sheet for staff questionnaires

Getting the Best from your NHS

Participant Information Sheet

We are undertaking an online health economics research study to measure the value that Patient Reported Outcome Measures (PROMs) add to NHS health services. A part of Value Based Healthcare is the completion of Patient Reported Outcome Measures (PROMs), which aim to guide professionals and services to deliver better care. We want to better understand how PROMs are impacting NHS services. Please read the below information to decide if you would like to take part.

**Introduction**

**Who are we?**

We are a team of experienced researchers at Bangor University in partnership with Aneurin Bevan University Health Board (ABUHB) Value Based Healthcare team.

**Why are we asking for your help?**

You are a professional involved in the delivery, set up or operationalising of Value Based Healthcare and Patient Reported Outcome Measures (PROMs). Your experience and perspectives are very important to help us measure the benefits that PROMs are adding to NHS services.

**How can I help?**

By completing this 20 minute online questionnaire.

**What does taking part involve?**

The online questionnaire will involve answering questions based on your experience from working in the NHS and from working with PROMs. All questions will be multiple choice and free text response.

**Who is organising and funding this study?**

Welsh Government are funding this study. Aneurin Bevan University Health Board are the sponsor.

**What are the possible benefits of taking part?**

Many people welcome the opportunity to share their views to help us learn. The information we gain will help us understand more about the use of Patient Reported Outcome Measures (PROMs) and the Value Based Healthcare programme.

**What are the possible disadvantages and risks of taking part?**

It is unlikely the questionnaire interview will cause distress, but rest assured you can stop at any point.

**What if I have concerns about this study?**

If you have a concern about any aspect of this study, you should ask to speak to a member of the research team who will do their best to answer your questions. The Chief Investigator is Dr Gareth Roberts, (email: hayley.lewis@wales.nhs.uk). You can also contact us on any of the channels provided at the end of this sheet. If the research team are unable to resolve your query and/or you would like to speak to somebody outside of the research team, you can contact the Aneurin Bevan Research manager on [ABB.RandD@wales.nhs.uk](mailto:ABB.RandD@wales.nhs.uk)

**Do I have to take part?**

No, it is entirely up to you.

**Research Contact details**

Ellena Crane: [e.crane@bangor.ac.uk](mailto:e.crane@bangor.ac.uk)

Dr Carys Jones: [c.l.jones@bangor.ac.uk](mailto:c.l.jones@bangor.ac.uk)

**Data protection information:**

**What will happen to the information I give?**

This questionnaire will be anonymous, and we will not ask you to provide any identifiable information (e.g. name, email). We will publish findings in academic articles, reports, and lay summaries. However, it will not be possible to identify you from any of the published findings.

**What are your choices about how your information is used?**

As a publicly funded organisation, we have to ensure that it is in the public interest when we use personally identifiable information from people who have agreed to take part in research. This means that when you agree to take part in a research study, we will use your data in the ways needed to conduct and analyse the research study. Your rights to access, change or move your information are limited, as we need to manage your information in specific ways in order for the research to be reliable and accurate. If you withdraw from the study, we will keep the information about you that we have already obtained. To safeguard your rights, we will use the minimum personally identifiable information possible.

You can find out more about how we use your information:

- https://www.hra.nhs.uk/information-about-patients/

- By asking the research team

- by contacting the Data Protection Officer Jonathan Meredith at [DPO.ABB@wales.nhs.uk](mailto:DPO.ABB@wales.nhs.uk).

Questionnaire for Clinical Staff in the Epilepsy Service

What is your role in the NHS?

- Doctor
- Nurse
- Allied healthcare professional
- PROMs co-ordinator
- Other (include free text box to describe_______)

---------------------------- Page Break ----------------------------

Based on a scoping review, stakeholder engagement and qualitative interviews, we have identified the main outcomes that PROMs can help achieve in Value-Based Healthcare.

Below is the list of outcomes that PROMs can help achieve. Please rank the outcomes based in order of what is **most important** to achieve within the epilepsy services **for patients** (with 1 being the highest priority).

*You can drag and drop to rearrange the statements below.*

[For this question item, we used the Ranking Question function from Survey Monkey]

| Patients being more informed, supported, and included in shared decision making about their epilepsy care |
| --- |
| Mental health problems in epilepsy patients being more efficiently and quickly identified |
| Patients in epilepsy service having quicker access to mental health support |
| Patient symptoms and changing needs being more quickly identified and acted upon |
| Patients being more confident in self-managing their health |
| Achieving more equitable access to care and treatment |
| More optimal care pathways in epilepsy care based on individual health needs |
| Better minimization of complications, unplanned admissions and unnecessary hospital visits related to epilepsy |
| More effective communication between healthcare staff |
| Improved service quality and performance |
| More efficient use of resources (i.e., finances, referrals, staff time, appointments) |

If you have further comments, or wish to expand upon your answers, please explain here.

Free text box _______

---------------------------- Page Break ----------------------------

In the next set of questions, we are interested in understanding to what extent these outcomes have been achieved in epilepsy services.

For each statement, tick the box that best represents the extent to which each outcome has been achieved within the ABUHB epilepsy services over the past 2 years.

1. Tick the box that best represents the extent the following outcome has been achieved within the ABUHB epilepsy services over the past 2 years.

Patients are more informed, supported, and included in shared decision making about their epilepsy care.

- *None of the time*
- *Rarely*
- *Often*
- *All of the time*
- *Not applicable*

*[Likert response options are repeated for all questions on this page]*

1. Tick the box that best represents the extent the following outcome has been achieved within the ABUHB epilepsy services over the past 2 years.

Mental health problems in epilepsy patients are more efficiently and quickly identified.

1. Tick the box that best represents the extent the following outcome has been achieved within the ABUHB epilepsy services over the past 2 years.

Patients in epilepsy service have quicker access to mental health support.

1. Tick the box that best represents the extent the following outcome has been achieved within the ABUHB epilepsy services over the past 2 years.

Patient symptoms and changing needs are more quickly identified and acted upon.

1. Tick the box that best represents the extent the following outcome has been achieved within the ABUHB epilepsy services over the past 2 years.

Patients are more confident in self-managing their health.

1. Tick the box that best represents the extent the following outcome has been achieved within the ABUHB epilepsy services over the past 2 years.

More equitable access to care and treatment.

1. Tick the box that best represents the extent the following outcome has been achieved within the ABUHB epilepsy services over the past 2 years.

More optimal care pathways in epilepsy care based on individual health needs.

1. Tick the box that best represents the extent the following outcome has been achieved within the ABUHB epilepsy services over the past 2 years.

Minimization of complications, unplanned admissions and unnecessary hospital visits related to epilepsy.

1. Tick the box that best represents the extent the following outcome has been achieved within the ABUHB epilepsy services over the past 2 years.

More effective communication between healthcare staff.

1. Tick the box that best represents the extent the following outcome has been achieved within the ABUHB epilepsy services over the past 2 years.

Improved service quality and performance.

1. Tick the box that best represents the extent the following outcome has been achieved within the ABUHB epilepsy services over the past 2 years.

More efficient use of resources (i.e., finances, referrals, staff time, appointments).

If you have further comments, or wish to expand upon your answers, please explain here.

Free text box _______

---------------------------- Page Break ----------------------------

The next set of questions is to find out **how much PROMs have contributed** to achieving the outcomes that you just answered questions on.

Below is the same list of outcomes. Please tick the box that best represents in your experience how much PROMs have helped in achieving each outcome over the past two years.

1. Tick the box that best represents how much PROMs have helped in achieving each outcome.

PROMs have helped patients be more informed, supported, and included in shared decision making about their epilepsy care.

- *Not at all*
- *Small amount*
- *Moderate amount*
- *Large amount*
- *Very large amount*
- *Not sure*
- *Not applicable*

*[Likert response options are repeated for all questions on this page]*

1. Tick the box that best represents how much PROMs have helped in achieving each outcome.

Because of PROMs, mental health problems in epilepsy patients are more efficiently and quickly identified.

1. Tick the box that best represents how much PROMs have helped in achieving each outcome.

Because of PROMs, patients in epilepsy service have quicker access to mental health support.

1. Tick the box that best represents how much PROMs have helped in achieving each outcome.

Because of PROMs, patient symptoms and changing needs are more quickly identified and acted upon.

1. Tick the box that best represents how much PROMs have helped in achieving each outcome.

PROMs help patients be more confident in self-managing their health.

1. Tick the box that best represents how much PROMs have helped in achieving each outcome.

Because of PROMs there is more equitable access to care and treatment.

1. Tick the box that best represents how much PROMs have helped in achieving each outcome.

Because of PROMs, there are more optimal care pathways in epilepsy care based on individual health needs.

1. Tick the box that best represents how much PROMs have helped in achieving each outcome.

PROMs help with minimizing complications, unplanned admissions and unnecessary hospital visits related to epilepsy.

1. Tick the box that best represents how much PROMs have helped in achieving each outcome.

PROMs help there be more effective communication between healthcare staff.

1. Tick the box that best represents how much PROMs have helped in achieving each outcome.

Because of PROMs, there is improved service quality and performance.

1. Tick the box that best represents how much PROMs have helped in achieving each outcome.

PROMs help with there being a more efficient use of resources (i.e., finances, referrals, staff time, appointments).

If you have further comments, or wish to expand upon your answers, please explain here.

Free text box _______

---------------------------- Page Break ----------------------------

To what extent do you think that patient mental health outcomes have **improved because of PROMs**?

- Not at all
- A small amount
- A moderate amount
- A large amount
- A very large amount

To what extent do you think that the use of PROMs has improved epilepsy care?

- Not at all
- A small amount
- A moderate amount
- A large amount
- A very large amount

Would it make any different to the care provided if PROMs were removed from the epilepsy care pathway?

- Not at all
- A small amount
- A moderate amount
- A large amount
- A very large amount

How much do you agree with the following statement.

‘The implementation of PROM questionnaires is sustainable in epilepsy care?’

*For this question, we are defining sustainable to mean that PROMs will continue to be delivered as intended and be integrated into routine practice following the initial implementation stage.*

- Strongly agree
- Agree
- Neither agree or disagree
- Disagree
- Strongly disagree

Can you think of an example(s) when PROMs have had an effect (either positive or negative) on the health or healthcare of a patient?

- Free text box _________

Questionnaire for Clinical Staff in the Heart Failure Service

What is your role in the NHS?

- Doctor
- Nurse
- Allied healthcare professional
- PROMs co-ordinator
- Other (include free text box to describe_______)

---------------------------- Page Break ----------------------------

Based on a scoping review, stakeholder engagement and qualitative interviews, we have identified the main outcomes that PROMs can help achieve in Value-Based Healthcare.

Below is the list of outcomes that PROMs can help achieve. Please rank the outcomes based in order of what is **most important** to achieve within the epilepsy services **for patients** (with 1 being the highest priority).

*You can drag and drop to rearrange the statements below.*

[For this question item, we used the Ranking Question function from Survey Monkey]

| Patients are better informed, supported, and included in shared decision making about their heart failure care. |
| --- |
| Patients are more confident in self-managing their health |
| Patient symptoms and changing needs are more quickly identified and acted upon (e.g., changing medication, referral to other services to address identified patient need) |
| Better patient health and life expectancy outcomes |
| More timely access to heart failure treatment for patients |
| Better minimization of complications, unplanned admissions and unnecessary hospital visits related to heart failure |
| More equitable access to care and treatment |
| More optimal care pathways in heart failure care based on individual health needs |
| More effective communication between healthcare staff |
| Improved service quality and performance in heart failure care |
| More efficient use of resources (i.e., finances, referrals, staff time, appointments) |

If you have further comments, or wish to expand upon your answers, please explain here.

Free text box _______

---------------------------- Page Break ----------------------------

In the next set of questions, we are interested in understanding to what extent these outcomes have been achieved in heart failure services.

For each statement, tick the box that best represents the extent to which each outcome has been achieved within the ABUHB heart failure services over the past 2 years.

1. Tick the box that best represents the extent the following outcomes have been achieved within the ABUHB heart failure services over the past 2 years.

Patients are more informed, supported, and included in shared decision making about their heart failure care.

- *None of the time*
- *Rarely*
- *Often*
- *All of the time*
- *Not applicable*

*[Likert response options are repeated for all questions on this page]*

1. Tick the box that best represents the extent the following outcomes have been achieved within the ABUHB heart failure services over the past 2 years.

Patients are more confident in self-managing their health.

1. Tick the box that best represents the extent the following outcomes have been achieved within the ABUHB heart failure services over the past 2 years.

Patient symptoms and changing needs are more quickly identified and acted upon (e.g., changing medication, referral to other services to address identified patient need).

1. Tick the box that best represents the extent the following outcomes have been achieved within the ABUHB heart failure services over the past 2 years.

Better patient health and life expectancy outcomes.

1. Tick the box that best represents the extent the following outcomes have been achieved within the ABUHB heart failure services over the past 2 years.

More timely access to heart failure treatment for patients.

1. Tick the box that best represents the extent the following outcomes have been achieved within the ABUHB heart failure services over the past 2 years.

Minimization of complications, unplanned admissions, and unnecessary hospital visits related to heart failure.

1. Tick the box that best represents the extent the following outcomes have been achieved within the ABUHB heart failure services over the past 2 years.

More equitable access to care and treatment.

1. Tick the box that best represents the extent the following outcomes have been achieved within the ABUHB heart failure services over the past 2 years.

More optimal care pathways in heart failure care based on individual health needs.

1. Tick the box that best represents the extent the following outcomes have been achieved within the ABUHB heart failure services over the past 2 years.

More effective communication between healthcare staff.

1. Tick the box that best represents the extent the following outcomes have been achieved within the ABUHB heart failure services over the past 2 years.

Improved service quality and performance in heart failure care.

1. Tick the box that best represents the extent the following outcomes have been achieved within the ABUHB heart failure services over the past 2 years.

More efficient use of resources (i.e. finances, referrals, staff time, appointments)

If you have further comments, or wish to expand upon your answers, please explain here.

Free text box _______

---------------------------- Page Break ----------------------------

The next set of questions is to find out **how much PROMs have contributed** to achieving the outcomes that you just answered questions on.

Below is the same list of outcomes. Please tick the box that best represents in your experience how much PROMs have helped in achieving each outcome over the past two years.

1. Tick the box that best represents how much PROMs have helped in achieving each outcome.

Because of PROMs, patients are more informed, supported, and included in shared decision making about their heart failure care.

- *Not at all*
- *Small amount*
- *Moderate amount*
- *Large amount*
- *Very large amount*
- *Not sure*
- *Not applicable*

*[Likert response options are repeated for all questions on this page]*

1. Tick the box that best represents how much PROMs have helped in achieving each outcome.

Because of PROMs, patients are more confident in self-managing their health.

1. Tick the box that best represents how much PROMs have helped in achieving each outcome.

Because of PROMs, patient symptoms and changing needs are more quickly identified and acted upon.

(e.g., changing medication, referral to other services to address identified patient need)

1. Tick the box that best represents how much PROMs have helped in achieving each outcome.

Because of PROMs, there are better patient health and life expectancy outcomes.

1. Tick the box that best represents how much PROMs have helped in achieving each outcome.

Because of PROMs, there is more timely access to heart failure treatment for patients.

1. Tick the box that best represents how much PROMs have helped in achieving each outcome.

PROMs have helped with minimizing complications, unplanned admissions and unnecessary hospital visits related to heart failure.

1. Tick the box that best represents how much PROMs have helped in achieving each outcome.

Because of PROMs, there is more equitable access to care and treatment.

1. Tick the box that best represents how much PROMs have helped in achieving each outcome.

Because of PROMs, there are more optimal care pathways in heart failure care based on individual health needs.

1. Tick the box that best represents how much PROMs have helped in achieving each outcome.

PROMs help there be more effective communication between healthcare staff.

1. Tick the box that best represents how much PROMs have helped in achieving each outcome.

Because of PROMs, there is improved service quality and performance.

1. Tick the box that best represents how much PROMs have helped in achieving each outcome.

PROMs help with there being a more efficient use of resources (i.e., finances, referrals, staff time, appointments).

If you have further comments, or wish to expand upon your answers, please explain here.

Free text box _______

---------------------------- Page Break ----------------------------

To what extent do you think that the use of PROMs has improved heart failure care?

- Not at all
- A small amount
- A moderate amount
- A large amount
- A very large amount

Would it make any difference to the care provided if PROMs were removed from the heart failure care pathway?

- Not at all
- A small amount
- A moderate amount
- A large amount
- A very large amount

1. How much do you agree with the following statement.

‘The implementation of PROM questionnaires is sustainable in heart failure care?’

*For this question, we are defining sustainable to mean that PROMs will continue to be delivered as intended and be integrated into routine practice following the initial implementation stage.*

- Strongly agree
- Agree
- Neither agree or disagree
- Disagree
- Strongly disagree

Can you think of an example(s) when PROMs have had an effect (either positive or negative) on the health or healthcare of a patient?

- Free text box _________

Questionnaire for Clinical Staff in the Parkinson’s Service

What is your role in the NHS?

- Doctor
- Nurse
- Allied healthcare professional
- PROMs co-ordinator
- Other (include free text box to describe_______)

---------------------------- Page Break ----------------------------

Based on a scoping review, stakeholder engagement and qualitative interviews, we have identified the main outcomes that PROMs can help achieve in Value-Based Healthcare.

Below is the list of outcomes that PROMs can help achieve. Please rank the outcomes based in order of what is **most important** to achieve within the epilepsy services **for patients** (with 1 being the highest priority).

*You can drag and drop to rearrange the statements below.*

[For this question item, we used the Ranking Question function from Survey Monkey]

| Patients being more informed, supported, and included in shared decision making about their Parkinson’s care. |
| --- |
| Patients being more confident in self-managing their health |
| Patient symptoms and changing needs being more quickly identified and acted upon (e.g., timely adaptation of medication) |
| Achieving more equitable access to care and treatment |
| More optimal care pathways based on individual health needs |
| Improved triage of patients into palliative care |
| Better minimization of complications, unplanned admissions, and unnecessary hospital visits related to Parkinson’s |
| More effective communication between healthcare staff |
| Improved service quality and performance in Parkinson’s care |
| More efficient use of resources (i.e., finances, referrals, staff time, appointments) |

If you have further comments, or wish to expand upon your answers, please explain here.

Free text box _______

---------------------------- Page Break ----------------------------

In the next set of questions, we are interested in understanding to what extent these outcomes have been achieved in Parkinson’s services.

For each statement, tick the box that best represents the extent to which each outcome has been achieved within the ABUHB Parkinson’s services over the past 2 years.

1. Tick the box that best represents the extent the following outcomes have been achieved within the ABUHB Parkinson’s services over the past 2 years.

Patients are more informed, supported, and included in shared decision making about their Parkinson’s care.

- *None of the time*
- *Rarely*
- *Often*
- *All of the time*
- *Not applicable*

*[Likert response options are repeated for all questions on this page]*

1. Tick the box that best represents the extent the following outcomes have been achieved within the ABUHB Parkinson’s services over the past 2 years.

Patients are more confident in self-managing their health.

1. Tick the box that best represents the extent the following outcomes have been achieved within the ABUHB Parkinson’s services over the past 2 years.

Patient symptoms and changing needs are more quickly identified and acted upon (e.g. timely adaptation of medication).

1. Tick the box that best represents the extent the following outcomes have been achieved within the ABUHB Parkinson’s services over the past 2 years.

More equitable access to care and treatment

1. Tick the box that best represents the extent the following outcomes have been achieved within the ABUHB Parkinson’s services over the past 2 years.

More optimal care pathways based on individual health needs.

1. Tick the box that best represents the extent the following outcomes have been achieved within the ABUHB Parkinson’s services over the past 2 years.

Improved triage of patients into palliative care.

1. Tick the box that best represents the extent the following outcomes have been achieved within the ABUHB Parkinson’s services over the past 2 years.

Better minimization of complications, unplanned admissions, and unnecessary hospital visits related to Parkinson’s.

1. Tick the box that best represents the extent the following outcomes have been achieved within the ABUHB Parkinson’s services over the past 2 years.

More effective communication between healthcare staff.

1. Tick the box that best represents the extent the following outcomes have been achieved within the ABUHB Parkinson’s services over the past 2 years.

Improved service quality and performance in Parkinson’s care.

1. Tick the box that best represents the extent the following outcomes have been achieved within the ABUHB Parkinson’s services over the past 2 years.

More efficient use of resources (i.e. finances, referrals, staff time, appointments)

If you have further comments, or wish to expand upon your answers, please explain here.

Free text box _______

---------------------------- Page Break ----------------------------

The next set of questions is to find out **how much PROMs have contributed** to achieving the outcomes that you just answered questions on.

Below is the same list of outcomes. Please tick the box that best represents in your experience how much PROMs have helped in achieving each outcome over the past two years.

1. Tick the box that best represents how much PROMs have helped in achieving each outcome.

Because of PROMs, patients are more informed, supported, and included in shared decision making about their Parkinson’s care.

- *Not at all*
- *Small amount*
- *Moderate amount*
- *Large amount*
- *Very large amount*
- *Not sure*
- *Not applicable*

*[Likert response options are repeated for all questions on this page]*

1. Tick the box that best represents how much PROMs have helped in achieving each outcome.

Because of PROMs, patients are more confident in self-managing their health.

1. Tick the box that best represents how much PROMs have helped in achieving each outcome.

Because of PROMs, patient symptoms and changing needs are more quickly identified and acted upon (e.g. timely adaptation of medication).

1. Tick the box that best represents how much PROMs have helped in achieving each outcome.

Because of PROMs, there is more equitable access to care and treatment.

1. Tick the box that best represents how much PROMs have helped in achieving each outcome.

Because of PROMs, there are more optimal care pathways based on individual health needs.

1. Tick the box that best represents how much PROMs have helped in achieving each outcome.

PROMs have helped with minimizing complications, unplanned admissions and unnecessary hospital visits related to heart failure.

1. Tick the box that best represents how much PROMs have helped in achieving each outcome.

Because of PROMs, there is more equitable access to care and treatment.

Because of PROMs, there is improved triage of patients into palliative care.

1. Tick the box that best represents how much PROMs have helped in achieving each outcome.

Because of PROMs, there are more optimal care pathways in heart failure care based on individual health needs.

PROMs have helped to minimize complications, unplanned admissions, and unnecessary hospital visits related to Parkinson’s.

1. Tick the box that best represents how much PROMs have helped in achieving each outcome.

PROMs help there be more effective communication between healthcare staff.

Because of PROMs, there is more effective communication between healthcare staff.

1. Tick the box that best represents how much PROMs have helped in achieving each outcome.

Because of PROMs, there is improved service quality and performance.

Because of PROMs, there is improved service quality and performance in Parkinson’s care.

1. Tick the box that best represents how much PROMs have helped in achieving each outcome.

PROMs help with there being a more efficient use of resources (i.e., finances, referrals, staff time, appointments).

If you have further comments, or wish to expand upon your answers, please explain here.

Free text box _______

---------------------------- Page Break ----------------------------

To what extent do you think that the use of PROMs has improved Parkinson’s care?

- Not at all
- A small amount
- A moderate amount
- A large amount
- A very large amount

Would it make any different to the care provided if PROMs were removed from the Parkinson’s care pathway?

- Not at all
- A small amount
- A moderate amount
- A large amount
- A very large amount

How much do you agree with the following statement.

‘The implementation of PROM questionnaires is sustainable in Parkinson’s care?’

*For this question, we are defining sustainable to mean that PROMs will continue to be delivered as intended and be integrated into routine practice following the initial implementation stage.*

- Strongly agree
- Agree
- Neither agree or disagree
- Disagree
- Strongly disagree

Can you think of an example(s) when PROMs have had an effect (either positive or negative) on the health or healthcare of a patient?

- Free text box _________

**Appendix 5:** Patient & Public Involvement

The study was developed with a wide range of Patient and Public Involvement (PPI) groups. These groups included individuals from Aneurin Bevan University Health Board and the Value-Based Healthcare Team at the health board, the third sector, and specific groups representing the services such as Aneurin Bevan Community Health Council, British Heart Foundation, Digital Communities Wales, Digital Wales, Epilepsy Action, Race Equality First, St. David’s Hospice Care, and the VBHC Patient Reference Group. A total of 10 virtual meetings were planned to address gaps, assist in the interpretation of findings, and ensure that outcomes were pertinent and accessible to the specific needs and circumstances of under-represented or vulnerable groups.

Various tasks, including conducting the scoping review, crafting patient questionnaires, and shaping initial program theories and logic models, were carried out with significant input from PPI contributors. Tailored sessions, that accounted for background and lived experience, were conducted for each group. All our PPI activities were guided by the UK standards for Public Involvement, and we used the GRIPP2 checklist to report and the PIRIT tool to track impact. Contributors were provided with expenses and financial reimbursement.

**Appendix 6:** Calculating Impact

**Table A2.** Evidence source & the number of stakeholders experiencing meaningful change for each outcome.

| **Outcome** | **Indicator & Source to Measure Outcome** | **Result from Indicator** | **Estimated number of Stakeholders that Experienced the Outcome.** |
| --- | --- | --- | --- |
| **Heart Failure** | | | |
| Improved management of patients heart failure leads to slightly better health and health outcomes. | Clinician Questionnaire Item:  Tick the box that best represents the extent the following *outcome has been achieved* within the ABUHB heart failure services over the *past 2 years.*  Better patient health and life expectancy outcomes. | 83% of participants answered ‘Often’ or ‘All of the time’ in response to this questionnaire item. | 923 / 1113 heart failure patients are estimated to have experienced this outcome in 2022. |
| PROMs help patients to be more knowledgeable, and confident in managing their heart failure. | Patient Questionnaire Item:  Please tick the box that best represents *your experiences* as a person with heart failure over the past two years. I have been better able to monitor my heart failure symptoms. | 38% of participants answered ‘Often’ or ‘All of the time’ in response to this questionnaire item. | 423 / 1113 heart failure patients are estimated to have experienced this outcome in 2022. |
| Triage of patients improves service efficiency and leads to more rapid access to care when needed. | Patient Questionnaire Item:  Please tick the box that best represents *your experiences* as a person with heart failure over the past two years. I have received more timely access to treatment and support for my heart failure.  Service Data  Average wait time for first appointment. | Patient Questionnaire Item:  34% of participants answered ‘Often’ or ‘All of the time’ in response to this questionnaire item.  Service Data  Prior to the introduction of PROMs in 2017/18, the average waiting time for a first appointment was 70 days. The three-year average waiting time for a first appointment between 2020 and 2023 was 22.6 days (SD = 6.0) Therefore, there has been a reduction in average wait time by 47.4 days. | 378 / 1113 heart failure patients are estimated to have experienced this outcome in 2022. |
| PROMs help patients feel more listened to and supported by their healthcare providers due to the provision of more patient-centred care. | Patient Questionnaire Item:  Please tick the box that best represents *your experiences* as a person with heart failure over the past two years.  In my heart failure care, my clinicians have listened to me more. | 49% of participants answered ‘Often’ or ‘All of the time’ in response to this questionnaire item. | 546 / 1113 heart failure patients are estimated to have experienced this outcome in 2022. |
| Reduced present demand on health service (reduced 1 FTE caseload). | Service Data  Administrative data on patient case load per 1 working time equivalent (WTE) per year.  Staff caseloads have reduced from 120 patients per 1 Working Time Equivalent (WTE) to 60 pts. This equates to a saving of 1 WTE per 60 patients. | Staff caseloads have reduced from 120 patients per 1 Working Time Equivalent (WTE) in 2017 prior to PROMs implementation to 60 patients per 1 WTE. This is a 50% reduction in patient caseload.  This enables staff to better manage patients with more complex healthcare needs. This has happened whilst there has been increased demand and is due to improved triage and discharge that streamlines patients to the healthcare pathway best suited to their needs.  There are currently 10.6 FTE nurses working in this service. To achieve the same outcome that has resulted from PROMs and pathway reform, an additional 10.6 FTE nurses would be needed to reduce patient caseloads by 50%. | 10 nurses (rounded down to avoid overclaiming) |
| **Epilepsy** | | | |
| Improved management of patients epilepsy leads to slightly better epilepsy-related health. | Patient Questionnaire Item:  Please tick the box that best represents your experiences in the past two years as a person with epilepsy.  My epilepsy has been more well controlled. | 39% of participants answered ‘Often’ or ‘All of the time’ in response to this questionnaire item. | 119 / 304 epilepsy patients are estimated to have experienced this outcome in 2022. |
| Due to improved identification and signposting to mental health support, patients have improved mental health. | Patient Questionnaire Item:  Please tick the box that best represents your experiences in the past two years as a person with epilepsy.  When mental health concerns have been identified by the epilepsy team, I have had quick access to mental health support. | 8% of participants answered ‘Often’ or ‘All of the time’ in response to this questionnaire item. | 12 / 304 epilepsy patients are estimated to have experienced this outcome in 2022. |
| PROMs help patients to be more knowledgeable and confident in managing their epilepsy and mental health. | Patient Questionnaire Item:  Please tick the box that best represents your experiences in the past two years as a person with epilepsy.  I have been better able to monitor my epilepsy symptoms. | 39% of participants answered ‘Often’ or ‘All of the time’ in response to this questionnaire item. | 119 / 304 epilepsy patients are estimated to have experienced this outcome in 2022. |
| PROMs help patients feel more listened to and supported by their healthcare providers due to the provision of more patient-centred care. | Patient Questionnaire Item:  Please tick the box that best represents your experiences in the past two years as a person with epilepsy.  In my epilepsy care, my clinicians have listened to me more. | 38% of participants answered ‘Often’ or ‘All of the time’ in response to this questionnaire item. | 116 / 304 epilepsy patients are estimated to have experienced this outcome in 2022. |
| **Parkinson’s** | | | |
| PROMs help patients to be more knowledgeable and confident in managing their Parkinson’s. | Patient Questionnaire Item:  Please tick the box that best represents *your experiences* as a person with Parkinson’s over the past two years.  I have been better able to monitor my Parkinson’s symptoms. | 46% of participants answered ‘Often’ or ‘All of the time’ in response to this questionnaire item. | 120 / 170 Parkinson’s patients are estimated to have experienced this outcome in 2022. |
| PROMs help patients feel more listened to and supported by their healthcare providers due to the provision of more patient-centred care. | Patient Questionnaire Item:  Please tick the box that best represents *your experiences* as a person with Parkinson’s over the past two years.  In my Parkinson’s care, my clinicians have listened to me more. | 71% of participants answered ‘Often’ or ‘All of the time’ in response to this questionnaire item. | 91 / 170 Parkinson’s patients are estimated to have experienced this outcome in 2022. |

**Table A3.** Evidence and methods used to determine the attribution for each outcome.

| **Outcome** | **Indicator & Source to Measure Attribution of Outcome to Other Variables** | **Result from Indicator** | **Estimated Attribution of Outcome to Other Variables** |  |  |
| --- | --- | --- | --- | --- | --- |
| **Heart Failure** | | | |  |  |
| Improved management of patients heart failure leads to slightly better health and health outcomes. | Clinician Questionnaire Item:  Tick the box that best represents how much PROMs have helped in achieving each outcome.  Because of PROMs, there are better patient health and life expectancy outcomes | 0% of participants answered ‘Large amount’ or ‘Very large amount’ in response to this questionnaire item. | 100% of this outcome is estimated to be attributed to other variables. |  |  |
| PROMs help patients to be more knowledgeable, and confident in managing their heart failure. | Patient Questionnaire Item:  Please tick the box that best represents how much PROMs helped in achieving this outcome.  PROMs have helped me to monitor my heart failure symptoms. | 25% answered ‘Large amount’ or ‘Very large amount’ in response to this questionnaire item. | 75% of this outcome is estimated to be attributed to other variables. |  |  |
| Triage of patients improves service efficiency and leads to more rapid access to care when needed. | Clinician Questionnaire Item:  Tick the box that best represents how much PROMs have helped in achieving each outcome.  Because of PROMs, there is more timely access to heart failure treatment for patients.  *We decided to use the clinician questionnaire item to measure attribution for the outcome, as we deemed it unlikely that patients will have insight into how useful PROMs are in achieving this outcome.* | 20% answered ‘Large amount’ or ‘Very large amount’ in response to this questionnaire item.  However, in consultation with clinician stakeholders it was recommended that the attribution to PROMs be increased by 10%. | 70% of this outcome is estimated to be attributed to other variables. |  |  |
| PROMs help patients feel more listened to and supported by their healthcare providers due to the provision of more patient-centred care. | Patient Questionnaire Item:  Please tick the box that best represents how much PROMs helped in achieving this outcome. In my heart failure care, PROMS have helped me in feeling more listened to by my clinician. | 25% answered ‘Large amount’ or ‘Very large amount’ in response to this questionnaire item. | 75% of this outcome is estimated to be attributed to other variables. |  |  |
| Reduced present demand on health service (reduced 1 FTE caseload). | Clinician Questionnaire Item:  Tick the box that best represents how much PROMs have helped in achieving each outcome.  Because of PROMs, there are better patient health and | 60% answered ‘Large amount’ or ‘Very large amount’ in response to this questionnaire item. | 40% of this outcome is estimated to be attributed to other variables. |  |  |
| **Epilepsy** | | | |  | **Epilepsy** |
| Improved management of patients epilepsy leads to slightly better epilepsy-related health. | Patient Questionnaire Item:  Please tick the box that best represents how much PROMs helped in achieving this outcome. PROMs have helped in better controlling my epilepsy. | 3% answered ‘Large amount’ or ‘Very large amount’ in response to this questionnaire item. | 97% of this outcome is estimated to be attributed to other variables. |  |  |
| Due to improved identification and signposting to mental health support, patients have improved mental health. | Patient Questionnaire Item:  Please tick the box that best represents how much PROMs helped in achieving this outcome. Because of PROMs, there are more optimal care pathways in heart failure care based on individual health needs | 0% answered ‘Large amount’ or ‘Very large amount’ in response to this questionnaire item. | 100% of this outcome is estimated to be attributed to other variables. |  |  |
| PROMs help patients to be more knowledgeable and confident in managing their epilepsy and mental health. | Patient Questionnaire Item:  Please tick the box that best represents how much PROMs helped in achieving this outcome. PROMs have helped me to monitor my epilepsy symptoms. | 6% answered ‘Large amount’ or ‘Very large amount’ in response to this questionnaire item. | 94% of this outcome is estimated to be attributed to other variables. |  |  |
| Triage of patients improves service efficiency and leads to more rapid access to care when needed. | Patient Questionnaire Item:  Please tick the box that best represents how much PROMs helped in achieving this outcome. PROMs have helped with my epilepsy treatment plan being more quickly adapted to changes in my health. | 0% answered ‘Large amount’ or ‘Very large amount’ in response to this questionnaire item. | 100% of this outcome is estimated to be attributed to other variables. |  |  |
| PROMs help patients feel more listened to and supported by their healthcare providers due to the provision of more patient-centred care. | Patient Questionnaire Item:  Please tick the box that best represents how much PROMs helped in achieving this outcome. In my epilepsy care, PROMS have helped in feeling more listened to by my clinician. | 6% answered ‘Large amount’ or ‘Very large amount’ in response to this questionnaire item. | 94% of this outcome is estimated to be attributed to other variables. |  |  |
| **Parkinson’s** | | | |  |  |
| PROMs help patients to be more knowledgeable and confident in managing their Parkinson’s. | Patient Questionnaire Item:  Please tick the box that best represents how much PROMs helped in achieving this outcome. PROMs help me to better monitor my Parkinson’s symptoms. | 30% answered ‘Large amount’ or ‘Very large amount’ in response to this questionnaire item. | 70% of this outcome is estimated to be attributed to other variables. |  |  |
| Triage of patients improves service efficiency and leads to more rapid access to care when needed. | Patient Questionnaire Item:  Please tick the box that best represents how much PROMs helped in achieving this outcome. PROMs have helped with my Parkinson’s treatment plan being more quickly adapted to changes in my health. | 10% answered ‘Large amount’ or ‘Very large amount’ in response to this questionnaire item. | 90% of this outcome is estimated to be attributed to other variables. |  |  |
| PROMs help patients feel more listened to and supported by their healthcare providers due to the provision of more patient-centred care. | Patient Questionnaire Item:  Please tick the box that best represents how much PROMs helped in achieving this outcome. In my Parkinson’s care, PROMs have helped me in feeling more listened to by my clinician. | 10% answered ‘Large amount’ or ‘Very large amount’ in response to this questionnaire item. | 90% of this outcome is estimated to be attributed to other variables. |  |  |

**Table A4.** Evidence and methods used to determine the displacement for each outcome.

| **Outcome** | **Indicator & Source to Measure Displacement** | **Result from Indicator** | **Estimated Displacement** |
| --- | --- | --- | --- |
| **Heart Failure** | | | |
| Improved management of patients heart failure leads to slightly better health and health outcomes. | Realist analysis & stakeholder involvement. | No evidence or reason to suggest any displacement effect. | 0% estimated displacement. |
| PROMs help patients to be more knowledgeable, and confident in managing their heart failure. | Realist analysis & stakeholder involvement. | No evidence or reason to suggest any displacement effect. | 0% estimated displacement. |
| Triage of patients improves service efficiency and leads to more rapid access to care when needed. | Realist analysis & stakeholder involvement. | There may be a small displacement effect of patients to other services. | 10% of patients are estimated to have shorter wait times due to displacement elsewhere within the NHS. |
| PROMs help patients feel more listened to and supported by their healthcare providers due to the provision of more patient-centred care. | Realist analysis & stakeholder involvement. | Realist analysis & stakeholder involvement. | No evidence or reason to suggest any displacement effect. |
| Reduced present demand on health service (reduced 1 FTE caseload). | Realist analysis & stakeholder involvement. | New PROMs based triage pathway was developed for patients discharged from acute cardiology care to the heart failure services. This reduced patient case load through (1) identifying inappropriate referrals and escalating to a consultant, or to another service improving overall NHS efficiency, and (2) through medical optimisation whereby patients are placed on the most appropriate care pathway and are more quickly discharged due to shorter waiting times and more efficient triage.  The majority of patients identified as inappropriate would have remained within the NHS given that they will have had health concerns that triggered the original referral. Although this pathway has increased efficiency, these patients are still displaced. For example, a third of patients are apparently referred to a cardiac rehabilitation program.  Using qualitative data and information from stakeholder invo, we estimated a 75% displacement rate for this outcome. | 75% of patients are estimated to be displaced elsewhere within the NHS. |
| **Epilepsy** | | | |
| Improved management of patients epilepsy leads to slightly better epilepsy-related health. | Realist analysis & stakeholder involvement. | Realist analysis & stakeholder involvement. | No evidence or reason to suggest any displacement effect. |
| Due to improved identification and signposting to mental health support, patients have improved mental health. | Realist analysis & stakeholder involvement. | There may be a small displacement effect of patients to other mental health services within the NHS. | 10% of patients are estimated to be displaced to other service within the NHS such as the Community Mental Health Team. |
| PROMs help patients to be more knowledgeable and confident in managing their epilepsy and mental health. | Realist analysis & stakeholder involvement. | Realist analysis & stakeholder involvement. | No evidence or reason to suggest any displacement effect. |
| PROMs help patients feel more listened to and supported by their healthcare providers due to the provision of more patient-centred care. | Realist analysis & stakeholder involvement. | Realist analysis & stakeholder involvement. | No evidence or reason to suggest any displacement effect. |
| **Parkinson’s** | | | |
| PROMs help patients to be more knowledgeable and confident in managing their Parkinson’s. | Realist analysis & stakeholder involvement. | Realist analysis & stakeholder involvement. | No evidence or reason to suggest any displacement effect. |
| PROMs help patients feel more listened to and supported by their healthcare providers due to the provision of more patient-centred care. | Realist analysis & stakeholder involvement. | Realist analysis & stakeholder involvement. | No evidence or reason to suggest any displacement effect. |

**Appendix 7:** Questionnaire Results

|  | *For items in Part 3 of the questionnaire* | *For items in Part 5 of the questionnaire* |
| --- | --- | --- |
| 1 | None of the time | Not at all |
| 2 | Rarely | Small amount |
| 3 | Some of the time | Moderate amount |
| 4 | Often | Large amount |
| 5 | All of the time | Very large amount |

**Table A6. Data analysis results from questionnaire for epilepsy patients**

| Questionnaire Item | Percentage of participants who answered >4 on the Likert Scale |
| --- | --- |
| Part 1 | |
| My epilepsy has been more well controlled. | 39% |
| My epilepsy treatment plan has been more quickly adapted to changes in my health. | 29% |
| Any mental health concerns have been more quickly identified by my epilepsy healthcare team. | 17% |
| When mental health concerns have been identified by the epilepsy team, I have had quick access to mental health support. | 8% |
| I have a better understanding and awareness of my epilepsy. | 42% |
| I have been better able to monitor my epilepsy symptoms. | 39% |
| In my epilepsy care, my clinicians have listened to me more. | 38% |
| I have felt more involved in making decisions about my epilepsy care. | 30% |
| Part 2 | |
| PROMs have helped in better controlling my epilepsy. | 3% |
| PROMs have helped with my epilepsy treatment plan being more quickly adapted to changes in my health. | 0% |
| PROMs have helped mental health concerns be more quickly identified by my epilepsy healthcare team. | 6% |
| PROMs have helped with getting quicker access to mental health support. | 0% |
| PROMs have help me to have a better understanding and awareness of my epilepsy. | 0% |
| PROMs have helped me to monitor my epilepsy symptoms. | 6% |
| In my epilepsy care, PROMS have helped in feeling more listened to by my clinician. | 6% |
| PROMs have helped in being involved in making decisions about my epilepsy care. | 6% |
| Overall, to what extent do you think PROMs improve your epilepsy care? | 7% |
| To what extent do you think that your mental health and/or mental health care improved because of PROMs? | 7% |
| Would it make any difference to you if PROMS were removed from your epilepsy care? | 10% |

**Table A7. Data analysis results from questionnaire for epilepsy clinical staff**

| Questionnaire Item | Percentage of participants who answered >4 on the Likert Scale |
| --- | --- |
| Part 1 | |
| Patients are more informed, supported, and included in shared decision making about their epilepsy care. | 80% |
| Mental health problems in epilepsy patients are more efficiently and quickly identified. | 60% |
| Patients in epilepsy service have quicker access to mental health support. | 40% |
| Patient symptoms and changing needs are more quickly identified and acted upon. | 80% |
| Patients are more confident in self-managing their health. | 0% |
| More equitable access to care and treatment. | 80% |
| More optimal care pathways in epilepsy care based on individual health needs. | 80% |
| Minimization of complications, unplanned admissions and unnecessary hospital visits related to epilepsy. | 40% |
| More effective communication between healthcare staff. | 80% |
| Improved service quality and performance. | 100% |
| More efficient use of resources (i.e., finances, referrals, staff time, appointments). | 20% |
| Part 2 | |
| PROMs have helped patients be more informed, supported, and included in shared decision making about their epilepsy care. | 60% |
| Because of PROMs, mental health problems in epilepsy patients are more efficiently and quickly identified | 40% |
| Because of PROMs, patients in epilepsy service have quicker access to mental health support | 40% |
| Because of PROMs, patient symptoms and changing needs are more quickly identified and acted upon | 60% |
| PROMs help patients be more confident in self-managing their health | 20% |
| Because of PROMs there is more equitable access to care and treatment | 40% |
| Because of PROMs, there are more optimal care pathways in epilepsy care based on individual health needs | 40% |
| PROMs help with minimizing complications, unplanned admissions and unnecessary hospital visits related to epilepsy | 0% |
| PROMs help there be more effective communication between healthcare staff | 20% |
| Because of PROMs, there is improved service quality and performance | 40% |
| PROMs help with there being a more efficient use of resources (i.e., finances, referrals, staff time, appointments) | 40% |
| To what extent do you think that the use of PROMs has improved epilepsy care? | 100% |
| To what extent do you think that the use of PROMs has improved mental health care? | 80% |
| Would it make any difference to the care provided if PROMs were removed from the epilepsy care pathway? | 80% |
| How much do you agree with the following statement.  ‘The implementation of PROM questionnaires is sustainable in epilepsy care?’ | 100% |

**Table A8. Data analysis results from questionnaire for heart failure patients**

| Questionnaire Item | *Percentage of participants who answered >4 on the Likert Scale* |
| --- | --- |
| Part 1 | |
| I have been better triaged to the best healthcare options based on my individual health and needs. | 33% |
| In my heart failure care, my clinicians have listened to me more. | 49% |
| I have felt more informed and supported in achieving any individual health goals I have with my heart failure. | 42% |
| I have felt more involved in making decisions about my heart failure. | 35% |
| I have a better understanding and awareness of heart failure and my individual symptoms. | 48% |
| I am able to monitor my heart failure symptoms. | 38% |
| I have felt more motivated to take action to look after my health. | 58% |
| I have received more timely access to treatment and support for my heart failure. | 34% |
| Changes or complications in my heart failure symptoms have been identified and acted upon more quickly by my healthcare team. | 35% |
| Part 2 | |
| PROMs have helped me in being better triaged to the best healthcare options based on my individual health and needs. | 28% |
| In my heart failure care, PROMS have helped me in feeling more listened to by my clinician. | 25% |
| PROMs have helped me in being more informed and supported in achieving any individual health goals I have with my heart failure. | 19% |
| PROMs have helped me in being involved in making decisions about my heart failure care. | 19% |
| PROMs have helped me to have a good understanding and awareness of heart failure and my individual symptoms. | 34% |
| PROMs have helped me to monitor my heart failure symptoms. | 25% |
| PROMs have helped me feel more motivated to take action to look after my health | 31% |
| PROMs have helped me to receive more timely access to treatment and support for my heart failure | 28% |
| PROMs have helped with changes or complications in my heart failure health being identified and acted upon more quickly. | 16% |
| Overall, to what extent do you think PROMs improve your heart failure care? | 62% |
| Would it make any difference to you if PROMS were removed from your heart failure care? | 47% |

**Table A9. Data analysis results from questionnaire for heart failure clinical staff**

| Questionnaire Item | *Percentage of participants who answered >4 on the Likert Scale* |
| --- | --- |
| Part 1 | |
| Patients are more informed, supported, and included in shared decision-making about their heart failure care. | 100% |
| Patients are more confident in self-managing their health. | 100% |
| Patient symptoms and changing needs are more quickly identified and acted upon (e.g. changing medication, referral to other services to address identified patient need). | 83% |
| Better patient health and life expectancy outcomes. | 83% |
| More timely access to heart failure treatment for patients. | 83% |
| More equitable access to care and treatment. | 50% |
| More optimal care pathways in heart failure care based on individual health needs. | 83% |
| More effective communication between healthcare staff. | 67% |
| Improved service quality and performance in heart failure care. | 83% |
| More efficient use of resources (i.e. finances, referrals, staff time, appointments). | 50% |
| Part 2 | |
| Because of PROMs, patients are more informed, supported, and included in shared decision making about their heart failure care. | 40% |
| Because of PROMs, patients are more confident in self-managing their health | 40% |
| Because of PROMs, patient symptoms and changing needs are more quickly identified and acted upon. | 20% |
| Because of PROMs, there are better patient health and life expectancy outcomes | 0% |
| Because of PROMs, there is more timely access to heart failure treatment for patients | 20% |
| Because of PROMs, there is more equitable access to care and treatment | 0% |
| Because of PROMs, there are more optimal care pathways in heart failure care based on individual health needs | 60% |
| Because of PROMs, there is more effective communication between healthcare staff | 20% |
| Because of PROMs, there is improved service quality and performance in heart failure care | 20% |
| Because of PROMs, there is more efficient use of resources (i.e., finances, referrals, staff time, appointments) | 20% |
| To what extent do you think that the use of PROMs has improved heart failure care? | 100% |
| Would it make any difference to the care provided if PROMs were removed from the heart failure care pathway? | 100% |
| How much do you agree with the following statement.  ‘The implementation of PROM questionnaires is sustainable in heart failure care?’ | 60% |

**Table A10. Data analysis results from questionnaire for Parkinson’s patients**

| Questionnaire Item | Percentage of participants who answered >4 on the Likert Scale |
| --- | --- |
| Part 1 | |
| In my Parkinson’s care, my clinicians have listened to me more. | 71% |
| I have felt more informed and supported in achieving any individual health goals I have with my Parkinson’s. | 42% |
| I have felt more involved in making decisions about my Parkinson’s care. | 50% |
| I have a better understanding and awareness of Parkinson’s and my individual symptoms. | 63% |
| I have been better able to monitor my Parkinson’s symptoms. | 46% |
| I have felt more motivated to take action to look after my health. | 58% |
| My Parkinson’s treatment plan has been more quickly adapted to changes in my health. | 42% |
| I have been better triaged to the best healthcare options based on my individual health and needs. | 46% |
| Part 2 | |
| In my Parkinson’s care, PROMs have helped me in feeling more listened to by my clinician. | 10% |
| PROMs have helped me in being more informed and supported in achieving any individual health goals I have with my Parkinson’s. | 20% |
| PROMs have helped me in being more involved in making decisions about my Parkinson’s care. | 20% |
| PROMs have helped me to have a better understanding and awareness of Parkinson’s and my individual symptoms. | 30% |
| PROMs help me to better monitor my Parkinson’s symptoms. | 30% |
| PROMs have helped me feel more motivated to take action to look after my health | 30% |
| PROMs have helped with my Parkinson’s treatment plan being more quickly adapted to changes in my health. | 10% |
| PROMs have helped me in being better triaged to the best healthcare options based on my individual health and needs. | 20% |
| Overall, to what extent do you think PROMs improve your Parkinson’s care? | 60% |
| Would it make any difference to you if PROMS were removed from your Parkinson’s care? | 50% |

**Table A11. Data analysis results from questionnaire for Parkinson’s clinical staff**

| Questionnaire Item | Percentage of participants who answered >4 on the Likert Scale |
| --- | --- |
| Part 1 | |
| Patients are more informed, supported, and included in shared decision making about their Parkinson’s care. | 67% |
| Patients are more confident in self-managing their health | 33% |
| Patient symptoms and changing needs are more quickly identified and acted upon (e.g. timely adaptation of medication) | 33% |
| More equitable access to care and treatment | 33% |
| More optimal care pathways based on individual health needs | 33% |
| Improved triage of patients into palliative care | 0% |
| Better minimization of complications, unplanned admissions, and unnecessary hospital visits related to Parkinson’s | 33% |
| More effective communication between healthcare staff | 33% |
| Improved service quality and performance in Parkinson’s care | 67% |
| More efficient use of resources (i.e., finances, referrals, staff time, appointments) | 33% |
| Part 2 | |
| Because of PROMs, patients are more informed, supported, and included in shared decision making about their Parkinson’s care. | 0% |
| Because of PROMs, patients are more confident in self-managing their health | 0% |
| Because of PROMs, patient symptoms and changing needs are more quickly identified and acted upon (e.g. timely adaptation of medication) | 0% |
| Because of PROMs, there is more equitable access to care and treatment | 0% |
| Because of PROMs, there are more optimal care pathways based on individual health needs | 0% |
| Because of PROMs, there is improved triage of patients into palliative care | 0% |
| PROMs have helped to minimize complications, unplanned admissions, and unnecessary hospital visits related to Parkinson’s | 0% |
| Because of PROMs, there is more effective communication between healthcare staff | 0% |
| Because of PROMs, there is improved service quality and performance in Parkinson’s care | 0% |
| Because of PROMs, there is more efficient use of resources (i.e., finances, referrals, staff time, appointments) | 0% |
| To what extent do you think that the use of PROMs has improved Parkinson’s care? | 33% |
| Would it make any difference to the care provided if PROMs were removed from the Parkinson’s care pathway? | 0% |
| How much do you agree with the following statement.  ‘The implementation of PROM questionnaires is sustainable in epilepsy care?’ | 33% |
